# Outcomes and prognostic factors in childhood-onset steroid-resistant nephrotic syndrome: a retrospective single-center study

**DOI:** 10.1007/s00467-025-06705-5

**Published:** 2025-03-01

**Authors:** Emil Zerkowitz, Jutta Gellermann, Juliane Beckus, Johannes Holle, Caroline Kempf, Philip Bufler, Dominik Müller, Julia Thumfart, Verena Klämbt

**Affiliations:** 1 Department of Pediatric Gastroenterology, Nephrology and Metabolic Diseases, Charité Universitätsmedizin, Berlin, Berlin, Germany; 2 Berlin Institute of Health, BIH Charité Clinician Scientist Program, Berlin, Germany

**Keywords:** Focal segmental glomerular sclerosis, FSGS, Proteinuria, Chronic kidney disease, Pediatric, SRNS

## Abstract

**Background:**

Steroid-resistant nephrotic syndrome (SRNS) is the second leading cause of chronic kidney disease (CKD) in childhood. It represents a heterogeneous group of diseases with variable kidney outcomes that are still challenging to predict. In this study, our main objective is to describe predictive factors of remission states and kidney survival comparing genetic and non-genetic SRNS.

**Methods:**

We conducted a retrospective analysis of 65 pediatric patients with SRNS treated at the pediatric outpatient clinic in Berlin between 2000 and 2023. Clinical characteristics, laboratory findings, and treatment strategies were systematically collected at multiple time points. Outcomes were defined by remission status, kidney survival (CKD stage I–IV), or progression to CKD stage V. Statistical analyses included univariate and multivariate logistic and Cox regression models adjusted for monogenic SRNS to identify predictors of remission and kidney survival.

**Results:**

The median age of onset was 4.0 years, with a male predominance of 57%. Patients were followed for a median of 5.9 years. At the last follow-up, 26 patients achieved complete remission, 12 achieved partial remission, and 27 showed no remission. Kidney survival rates at 5 and 10 years were 71% and 56%, respectively. High initial nephrotic-range proteinuria, confirmed genetic diagnoses, reduced eGFR, and hypoalbuminemia at 3-month and 1-year follow-ups were identified as negative predictive factors for complete or partial remission. These factors also correlated strongly with an elevated risk of progression to CKD stage V.

**Conclusion:**

Our findings highlight additional prognostic factors influencing remission status and long-term kidney survival in pediatric SRNS, emphasizing the value of detailed early time-point analyses.

**Graphical abstract:**

**Figure:**
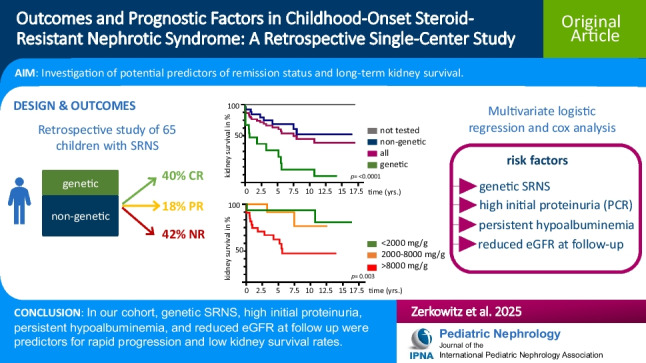
A higher resolution version of the Graphical abstract is available as Supplementary information

**Supplementary Information:**

The online version contains supplementary material available at 10.1007/s00467-025-06705-5.

## Introduction

Nephrotic syndrome (NS) is defined as a combination of nephrotic-range proteinuria (protein–creatinine ratio (PCR) > 2000 mg/g), hypoalbuminemia (< 30 g/l), and edema [1, 2]. The majority of childhood-onset NS cases are idiopathic, while secondary causes, resulting from systemic diseases, infections, or toxic exposures, are relatively rare. Idiopathic NS can be further classified based on the response to standard oral corticosteroid therapy. Approximately 85% of cases achieve remission within 6 weeks, indicating steroid-sensitive NS (SSNS), with those achieving complete remission between 4 and 6 weeks classified as late responders. Patients who do not respond after 6 weeks are diagnosed with steroid-resistant NS (SRNS), which is associated with significantly poorer kidney outcomes [3, 4]. Some patients with SSNS may also develop secondary SRNS in subsequent relapses, defined as secondary SRNS, accounting for 13.8–36% of cases [5–7]. These patients have a reported 10-year kidney survival rate of 85.8% [5], but an increased risk of post-transplant recurrence [8]. SRNS presents the second leading cause of chronic kidney disease (CKD) in childhood, with 36–57% of patients progressing to CKD stage V within 10 years of diagnosis [9–11]. SRNS is commonly associated with focal segmental glomerulosclerosis (FSGS), although other histological patterns, such as minimal change disease (MCD) and diffuse mesangial sclerosis (DMS), may also be observed. Pathogenic disease-causing genetic variants are identified in 29% of SRNS cases [12]. In patients with a positive family history, consanguinity, or infantile onset, a monogenic causation can be detected in up to 69% [12–14]. Patients with monogenic SRNS typically do not respond to immunosuppressive treatment. Therefore, genetic testing helps avoid unnecessary exposures to immunosuppressants, guides post-transplant management, and enables personalized treatment options on a gene-specific basis (e.g., coenzyme Q supplementation) [15, 16]. According to the current clinical practice guidelines established by the International Pediatric Nephrology Association (IPNA), patients who fail to achieve complete remission after 6 weeks of oral corticosteroid therapy are considered steroid-resistant and should be evaluated with kidney biopsy and genetic testing [3]. The IPNA guidelines do not recommend genetic testing in cases of secondary SRNS. The treatment should include renin–angiotensin–aldosterone system (RAAS) blockers combined with ongoing oral prednisolone potentially supplemented with methylprednisolone pulses. Additionally, patients should receive intensified immunosuppressive (IIS) therapy, typically involving calcineurin inhibitors. The IIS therapy plan should be re-evaluated upon receipt of genetic testing results [3]. Concurrently, a tapering regimen for oral corticosteroids is advised to reduce the risk of long-term steroid-related side effects [3].

The course of SRNS is highly variable and remains challenging to predict due to significant heterogeneity in disease pathophysiology. For many years, kidney biopsy–based histopathological classifications have been the primary tool for SRNS stratification; however, their predictive accuracy for long-term kidney outcomes remains limited. Recent research has explored the roles of genetic factors, response to immunosuppression, age at disease onset, hematuria, persistent proteinuria, hypertension, and other potential modifiers to refine predictions of kidney outcomes and remission status [9, 10, 17–19]. However, only Trautmann et al. [10] have accounted for monogenic SRNS as predictor while fully adjusting for potential confounders. In this single-center, retrospective study, we analyze clinical, biochemical, and treatment parameters with the main objective to describe prediction factors of remission status and kidney survival adjusted for genetic SRNS in a well-defined local pediatric SRNS cohort at multiple time points.

## Methods

### Patient cohort and retrospective data analysis

**Fig. 1:**
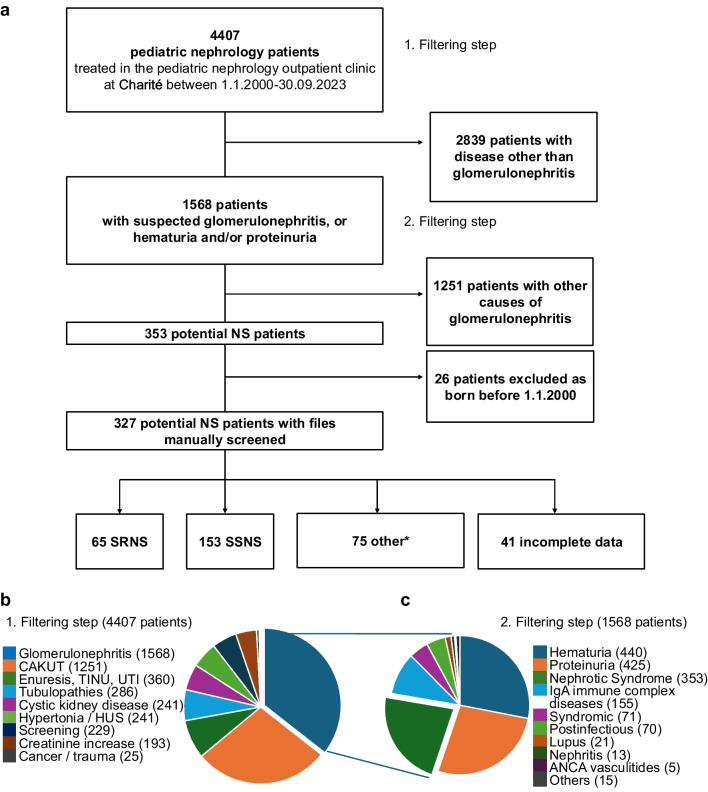
Screening process for SRNS identification (**a**) Flowchart illustrating the stepwise screening to identify SRNS patients within all patients treated at the KfH pediatric nephrology outpatient clinic between 2000 and 2023. **b** 4407 patients were initially prescreened into 9 categories depending on their initial diagnosis. **c** The following initial diagnoses were subgrouped into “glomerulonephritis” cohort (Supplemental Table 1)

In this single-center, retrospective cohort study, data from 4407 patients treated at the Kuratorium fuer Dialyse und Nierentransplantation (KfH) pediatric nephrology outpatient clinic located in Berlin between January 2000 and September 2023 were systematically filtered to identify potential SRNS cases. A flowchart outlining the stepwise screening process for identifying patients with SRNS is provided in Fig. 1a (Supplemental Table 1). Patients meeting the following criteria were included:

1. A confirmed diagnosis of NS with either primary or secondary resistance to oral corticosteroid therapy; primary steroid resistance was defined as failure to respond to standard oral corticosteroid therapy after 6 weeks ([3]).
2. Age at diagnosis between 0 and 18 years.

**Table 1:** Patient characteristics of 65 SRNS patients at initial presentation and follow-up. Data is presented in total numbers or as median (range in brackets)

Patient characteristics		Total *n* = 65	Genetic *n* = 19	Non-genetic *n* = 33	Not tested *n* = 13	*p*-value	CR *n* = 26	PR *n* = 12	NR *n* = 27	*p*-value
Demographics										
Gender	Female Male	28 37	12 7	11 22	5 8	0.105	11 15	3 9	14 13	0.293
Ethnicity	European Middle eastern Mixed	40 22 3	10 8 1	25 7 1	5 7 1	*0.575	18 6 2	9 3 0	13 13 1	*0.1038
Consanguinity	Yes No Unknown	9 52 4	7 12 0	1 32 0	1 8 4	*0.002	0 23 3	1 10 1	8 19 0	* < 0.001
Positive family history		6	6	0	0	* < 0.001	1	2	3	0.686
Age of onset	Median (mths) (range) 0–3 months > 3–12 months > 1–6 years > 6–12 years > 12–18 years	47.47 (0–213) 3 3 38 15 6	34.70 (0.4–119) 2 3 10 4 0	57.87 (0–190.8) 1 0 20 10 2	42.73 (14.2–213.3) 0 0 8 1 4	**0.042** *0.016	50.90 (17.3–213.2) 0 0 17 5 4	50.03 (14.2–208.1) 0 0 7 4 1	38.60 (0–169.7) 3 3 14 6 1	0.093 *0.034
Histology	FSGS MCD DMS Other Normal missing data	41 12 4 1 2 5	11 0 3 1 1 3	25 5 0 0 1 2	5 7 1 0 0 0	*0.003	16 9 1 0 0 0	9 3 0 0 0 0	16 0 3 1 2 5	* < 0.001
Steroid resistance	Primary Secondary	58 7	18 1	29 4	11 2	*0.663	21 5	10 2	27 0	*0.036
Syndromic features		12	9	3	0	*0.003	2	2	8	*0.061
Initial presentation										
Initial laboratory results	Albumin (S) g/l Prot./Crea (U) mg/g	23.10 7022	25.50 17,420	21.70 6774	21.00 2444	0.630 0.059	21.10 2800	25.40 6650	21.35 13,379	0.130 ** < 0.001**
Initial kidney function	eGFR median 1 2 3a 3b 4 5 Dialysis	123.18 44 8 3 2 0 2 2	94.30 9 5 2 1 0 1 1	148.54 24 1 1 1 0 1 1	131.15 11 2 0 0 0 0 0	0.110 *0.036	156.73 24 1 0 0 0 0 0	118.47 9 3 0 0 0 0 0	94.30 11 4 3 2 0 2 2	**0.015** * < 0.001
Follow-up information										
Multiple drugs within 1 year		29	4	18	7	**0.049**	11	5	13	0.889
Kidney failure	Yes Dialysis Transplanted Deceased Median time to CKD V in months (range)	23 6 14 3 11.63 (0–196.8)	14 4 7 3 7.37 (0–162.00)	9 2 7 0 27.97 (0.13–196.80)	0 0 0 0	** < 0.001** 0.394	0 0 0 0	0 0 0 0	23 6 14 3 11.63 (0–196.8)	** < 0.001**
Observation period in years	Median (Range)	5.85 (0.2–18.0)	3.90 (0.7–18.0)	7.54 (0.2–16.4)	5.25 (0.9–14.1)	0.682	5.55 (0.7–16.4)	5.94 (0.8–9.0)	5.91 (0.2–18.0)	0.406

Patients are divided into subgroups according to their final genetic and remission status. Proteinuria, Hypalbuminemia, eGFR, and relapses are listed as Supplemental Table 3. *FSGS*, focal segmental glomerulosclerosis; *MCD*, minimal change disease; *DMS*, diffuse mesangial sclerosis; *other*, diffuse glomerular mesangial matrix expansion without sclerosis); *CR*, complete remission; *PR*, partial remission; *NR*, no remission, *Crea S*, serum creatinine; *Prot/Crea U*, spot urine protein to creatinine ratio; *eGFR*, estimated glomerular filtration rate, in ml/min; *CKD*, chronic kidney disease. ​*p*-values are calculated by chi-squared for categorical values and the Kruskal–Wallis test for continuous variables. *p-value calculated using Fisher’s exact test after combining

Exclusion criteria were as follows:

1. Patients born before January 1, 2000
2. Cases of secondary SRNS causes
3. Patient files lacking at least one follow-up data entry

Epidemiological characteristics such as gender, ethnicity, family history, and age of onset were collected from patients’ initial clinical records. Clinical data, such as type of steroid resistance, syndromic features, biopsy results, or administered medication, were collected from medical records. Patients underwent genetic testing either through commercially available SRNS gene panel sequencing or whole-exome sequencing. The genes included in the panel varied over time. The patients’ genetic test results were used to classify patients into genetic or non-genetic subgroups. Biochemical data including serum creatinine, serum albumin, and urine protein–creatinine ratio (PCR) as well as anthropometric measurements (weight and height) were recorded at specific time points when available: the initial presentation, 3 months, 12 months, 2 years, 3 years, 5 years, and 10 years. Additionally, a last follow-up visit was recorded. If PCR results were unavailable, they were estimated using urine dipstick results [20] or the protein measurements from 24-h urine collections. Estimated glomerular filtration rate (eGFR) was calculated using the Schwartz formula [21].

### Outcome measures

The primary outcomes included complete remission, partial remission, no remission, kidney survival, and kidney failure. Remission status was defined according to current KDIGO criteria [22]:

- **Complete remission**: PCR < 0.2 g/g, or negative/trace dipstick in first void urine on three consecutive days.
- **Partial remission**: ≥ 50% reduction of proteinuria with PCR between 0.2 and 2 g/g and serum albumin ≥ 30 g/l.
- **No remission**: Failure to meet remission criteria.
- **Kidney survival**: Defined as CKD stage I–IV.
- **Kidney failure**: CKD stage V (eGFR < 15 ml/min), including patients with CKD stage VD and death.

### Statistical analysis

All statistical analyses were performed using GraphPad Prism version 10.3.1 for Windows (GraphPad Software, Boston, MA, USA). Due to limited sample size, a non-normal distribution was assessed. Median and range values were calculated and statistical significance set at *p* ≤ 0.05. For categorical variables, the chi-squared test was used. Fisher’s exact test was applied when chi-squared test was invalid due to small sample sizes after combining subgroups complete and partial remission, or non-genetic and untested groups. For continuous variables, the Kruskal–Wallis test was used to identify differences between groups.

The odds ratio (OR) for a favorable remission outcome—defined as partial or complete remission at the last follow-up—was calculated using univariate logistic regression and is reported with 95% confidence intervals (CI). All significant variables identified in the univariate analysis were included in the multivariate logistic regression model, excluding multiple drug variables. Cox regression analysis was conducted to obtain hazard ratios (HR) for progression to kidney failure. An additional Cox regression model, adjusted for genetic disease, was used to account for potential confounding. Significant variables identified from the adjusted Cox model were included in a multiple Cox regression analysis to further limit confounding effects. The Kaplan–Meier analysis was used to calculate kidney survival rates.

## Results

### Identification of patient cohort

A total of 4407 patients were treated at our pediatric nephrology outpatient clinic between January 2000 and September 2023 (Fig. 1a). One thousand five hundred sixty-eight patients presented with symptoms associated with glomerulonephritis (Fig. 1b, c). Among those, 353 potential NS cases were identified (Fig. 1c, Supplemental Table 1). After excluding 26 patients born before 2000, 327 cases were reviewed individually. Of these, 153 were classified as SSNS based on oral corticosteroid response, 65 as steroid-resistant, 75 were reclassified, and 41 lacked follow-up data (Fig. 1a).

### Demographics and clinical features of SRNS cases

Within the cohort of 65 SRNS cases, the mean age of disease onset was 4.0 years (range 0–17.8 years) (Table 1). The cohort comprised 37 males (57%) and 28 females (43%). Pathogenic variants in SRNS-associated genes were identified in 19 cases (29%) (Supplemental Table 2). Negative genetic screening results were observed in 33 patients (51%), while genetic data was unavailable for 13 patients (20%) (Table 1). Syndromic features were significantly more frequent in the genetic cohort compared to the non-genetic cohort (*p* = 0.003). Consanguinity was reported in 9 cases: 7 cases in the genetic cohort, 1 case in the non-genetic cohort, and 1 case in the untested group (*p* = 0.002). Additionally, 6 patients—all in the genetic cohort—had a positive family history (*p* < 0.001). Relapses varied between groups. They occurred more frequently in the non-genetic (7 out of 33 patients, 1–12 relapses) and the untested group (8 out of 13 patients, 1–13 relapses) compared to the genetic group (2 out of 19, 1–3 relapses, *p* = 0.011) (Supplemental Table 3). Kidney biopsies were performed in 60 patients (92%), revealing FSGS as the most common finding (68%) (Table 1). Among the 65 SRNS cases, 58 (89%) exhibited primary steroid resistance, while 7 (11%) demonstrated secondary steroid resistance including one patient with a genetic variant. The median follow-up period for all patients was 5 years and 10 months.

**Table 2:** Immunosuppressive treatment (IIS) administered to all SRNS patients following initial diagnosis

Treatment		Total *n* = 65	CR *n* = 26	PR *n* = 12	NR *n* = 27
Steroid pulse	3 × 500 mg/m^2^	46	22	10	14
Oral IIS	CsA Tacrolimus MMF CNI + MMF	52 (8.93) 8 (14.96) 30 (37.00) 33 (7.60)	26 (12.53) 2 (31.86) 21 (41.20) 18 (8.83)	12 (10.40) 2 (17.70) 4 (39.10) 5 (6.50)	14 (5.40) 4 (5.37) 5 (7.63) 10 (7.23)
Rituximab*		5	1 (2 ×)	0	4 (1–5 ×)
Cyclophosphamide		4	3	0	1
Indomethacin		4 (15.47)	0	0	4 (15.47)
RAAS inhibitors	ACE inhibitors	57 (42.07)	20 (38.87)	12 (31.12)	25 (50.47)
Nephrectomy		9	0	0	9

Total number of patients are documented; the median duration of prescription is indicated in month in brackets (). *CsA*, cyclosporine A; *MMF*, mycophenolate mofetil; *CNI*, calcineurin inhibitor; *RAAS*, renin–angiotensin–aldosterone system; *ACE*, angiotensin converting enzyme; *CR*, complete remission; *PR*, partial remission; *NR*, no remission ​

**Table 3:** Predictors for remission (partial and complete combined) and CKD stage V. Each value was calculated individually as univariate logistic regression for odds ratio (OR) and Cox regression for hazard ratio (HR)

Patient characteristics	Remission (CR + PR)				CKD Stage V					
	Univariate logistic		Multivariate logistic		Cox regression		Adjusted Cox regression		Multiple Cox regression	
	*p-*value	OR (95% COI)	*p-*value	OR (95%COI)	*p-value*	HR (95%COI)	*p-value*	HR (95%COI)	*p-value*	HR (95%COI)
Gender (ref. female)	0.155	2.08 (0.76–5.83)			0.077	0.43 (0.16–1.07)	0.221	0,56 (0.21–1.40)		
Age of onset (ref. > 6 years) < 1 year 1–6 years	0.053	1.01 (1.00–1.03)			0.158 **0.014** 0.668	0.99 (0.98–1.00) 4.86 (1.33–17.87) 0.78 (0.27–2.59)	0.721 0.492 0.721	1.00 (0.98–1.01) 1.60 (0.41–6.37) 0.82 (0.28–2.67)		
Consanguinity	**0.014**	0.067 (0.01–0.40)	0.584	0.46 (0.02–6.85)	** < 0.001**	7.19 (2.54–19.33)	**0.007**	4.37 (1.45–12.74)	0.140	4.27 (0.51–30.00)
Family history	0.701	0.72 (0.12–4.19)			0.094	3.07 (0.68–10.30)	0.845	0.87 (0.19–3.15)		
Syndromic	0.159	0,40 (0.11–1.42)			**0.007**	3.79 (1.37–9.25)	0.424	1.52 (0.53–4.2)		
Initial proteinuria (PCR) (ref. < 2000) 2000–8000 > 8000	**0.035** **0.027** ** < 0.001**	1.00 (0.99–1.00) 6.90 (1.67–47.33) 0.10 (0.02–0.32)	0.887 0.561 **0.035**	1.00 (1.00–1.00) 0.41 (0.01–8.01) 0.04 (0.01–0.52)	** < 0.001** **0.024** **0.016**	1.00 (1.00–1.00) 0.18 (0.03–0.65) 0.16 (0.03–0.58)	0.134 0.503 **0.020**	1.00 (1.00–1.00) 2.06 (0.22–19.02) 6.86 (1.69–48.80)	**0.031**	4.98 (1.25–25.87)
Biopsy (ref. MCD) FSGS DMS	0.202 0.090	0.40 (0.08–1.49) 0.13 (0.01–1.12)			> 0.999 > 0.999		> 0.999 > 0.999			
Multiple drugs	**0.011**	0.12 (0.02–0.52)	+		0.106	3.62 (0.90–24.07)	**0.032**	6.27 (1.40–46.15)	0.064	4.97 (1.07–37.70)
Initial eGFR > 90	**0.019** **0.002**	1.01 (1.00–1.02) 8.50 (2.40–36.01)	0.394 0.683	1.01 (0.99–1.03) 1.86 (0.08–38.29)	**0.006** ** < 0.001**	0.98 (0.98–1.00) 0.19 (0.07–0.49)	0.155 0.133	0.99 (0.98–1.01) 0.45 (.016–1.25)		
eGFR decline (within 1 year)	0.910	1.00 (1.00–1.01)			0.864	1.00 (0.99–1.01)	0.740	1.00 (0.99–1.01)		
+ Genetic diagnosis	** < 0.001**	0,08 (0.02–0.28)	**0.030**	0.09 (0.01–0.69)	** < 0.001**	8.19 (3.21–23.47)			**0.014**	5.60 (1.35–23.39)
Drug resistance = NR after 1 year	0.719	1.20 (0.44–3.34)			0.905	0.95 (0.37–2.43)	0.126	2.29 (0.80–6.84)		
Follow-up information										
Proteinuria at 3 months	+		+		+		+		+	
Hypoalbuminemia at 3 months	0.103	0.37 (0.10–1.20)	**0.015**	0.14 (0.03–0.61)	0.203	2.12 (0.67–7.26)	**0.033**	4.02 (1.15–15.58)	**0.020** *0.009	5.69 (1.39–28.32) *18.56 (2.81–264.62)
eGFR at 3 months < 90	**0.009**	1.02 (1.01–1.04)	**0.003**	1.03 (1.01–1.05)	**0.001** 0.081	0.97 (0.95–0.99) 2.76 (0.86–8.90)	**0.009** 0.117	0.98 (0.96–0.99) 2.55 (0.77–8.49)	** < 0.001** *0.002	0.96 (0.93–0.98) *0.97 (0.94–0.99)
Proteinuria at 1 year	**0.045**	0.11 (0.01–0.67)	0.153	0.18 (0.01–1.46)	0.252	3.38 (0.61–62.92)	0.129	5.1 (0.91–95.75)	0.474 *0.608	2.37 (0.27–50.77) *1.93 (0.18–45.54)
Hypoalbuminemia at 1 year	**0.010**	0.14 (0.03–0.60)	**0.046**	0.15 (0.02–0.87)	0.084	3.24 (0.85–13.26)	**0.009**	20.52 (2.95–420.3)	0.146 *0.010	3.20 (0.71–18.64) *48.3 (4.01–1969.22)
eGFR at 1 year < 90	0.102	1.01 (1.00–1.03)	**0.033**	1.02 (1.01–1.04)	0.058 **0.039**	0.98 (0.97–1.00) 5.27 (1.27–35.50)	0.272 **0.031**	0.99 (0.97–1.01) 6.84 (1.43–54,88)	**0.020** *0.105	0.98 (0.96–0.99) *0.98 (0.95–1.00)

All significant values were included for multivariate logistic and multivariate Cox regression. Furthermore, a Cox regression adjusted for genetic disease was performed. For follow-up information, the multivariate analysis included all values of the respective observation point. ​Significant results are in bold. ​ + excluded from multivariate regression due to perfect separation. ​ *Additionally adjusted for genetic disease

### Treatment

**Fig. 2:**
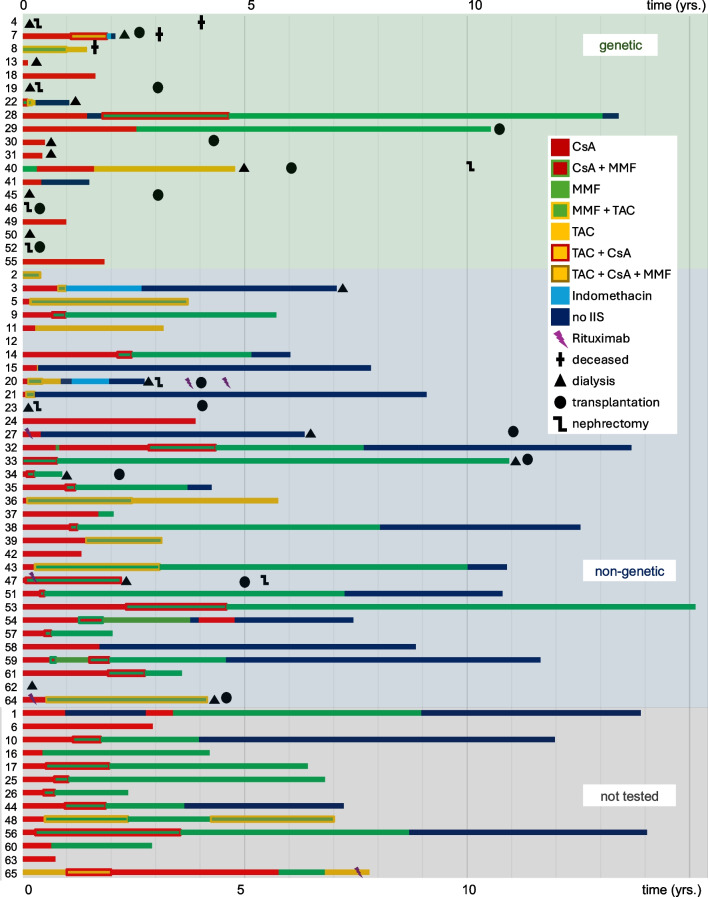
Timeline of individually administered medications and kidney outcomes for all patients, sorted by their genetic testing results. CsA, cyclosporine A; MMF, mycophenolate mofetil; TAC, tacrolimus; IIS, intensified immunosuppression

During the first year of treatment, 56 of 65 patients received intensified immunosuppressive (IIS) therapy, including 43 with steroid pulses, 52 with cyclosporine A (CsA), 2 with tacrolimus, 10 with mycophenolate mofetil (MMF), and 23 with a combination of CNI and MMF (Supplement Table 4). The patients not receiving IIS, directly underwent dialysis (*n* = 6), were kidney transplanted (*n* = 2) or did not receive any treatment due to incompliance (*n* = 1) (Fig. 2). Changes to immunosuppressive regimens were made in 29 patients during the first year of treatment; occurring more frequently in the non-genetic (55%) and untested group (54%) compared to the genetic group (21%) (*p* = 0.049).

Over the entire observation period, 52 patients received CsA, 8 were treated with tacrolimus, 30 with MMF, and 33 received a combination of CNI and MMF. Nine patients underwent nephrectomy due to severe, treatment-resistant proteinuria after initiating dialysis or prior kidney transplantation (Table 2, Fig. 2). Individual treatment protocols of each patient stratified according to genetic testing result are shown in Fig. 2, while treatments, grouped by final remission response, are listed individually in Supplemental Fig. 1.

### Remission status

**Fig. 3:**
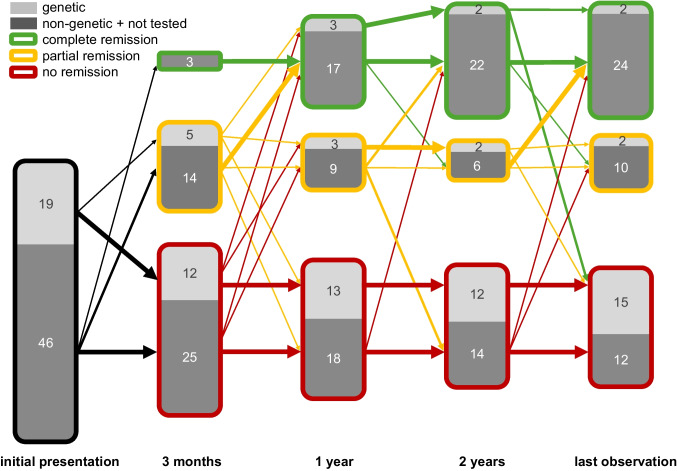
Distribution of genetic and non-genetic cases within remission subgroups. Arrow thickness indicates the relative amount of movement in between groups (CR, PR, and NR). All movements within the first 2 years and at the time of last observation are displayed

Within the first year, 32 patients (51%) achieved complete or partial remission (CR or PR), while 31 patients (49%) remained in no remission (NR) (Fig. 3). At last follow-up, 26 patients (40%) were in CR, 12 patients (18%) were in PR, and 27 (42%) were in NR (Fig. 3). The most frequent transition between remission groups was from PR to CR (Fig. 3, Supplemental Table 5). Significant differences in initial eGFR and PCR were observed among the final remission groups (Table 1). The median eGFR at first presentation varied significantly between CR, PR, and NR groups, with the highest median eGFR in the CR group and the lowest in the NR group (*p* = 0.015, Supplemental Table 3). Median initial PCR values were 2800 mg/g (range 0–18,971 mg/g) in the CR group, 6650 mg/g (range 520–95,944 mg/g) in the PR group, and 13,379 mg/g (range 780–152,380 mg/g) in the NR group (*p* < 0.001, Table 1). Notably, consanguinity (*p* < 0.001) and primary steroid resistance (*p* = 0.036) were significantly more frequent in the NR group (Table 1). Among patients with genetic SRNS, 15 of 19 patients (79%) did not achieve remission at last follow-up, accounting for 56% of entire NR group (27 patients). Four patients with a disease-causing variant (Supplemental Table 6) achieved CR or PR following CsA treatment. Among these, two patients with PR exhibited secondary steroid resistance, while the two patients with CR demonstrated primary steroid resistance.

### Kidney survival and CKD

**Fig. 4:**
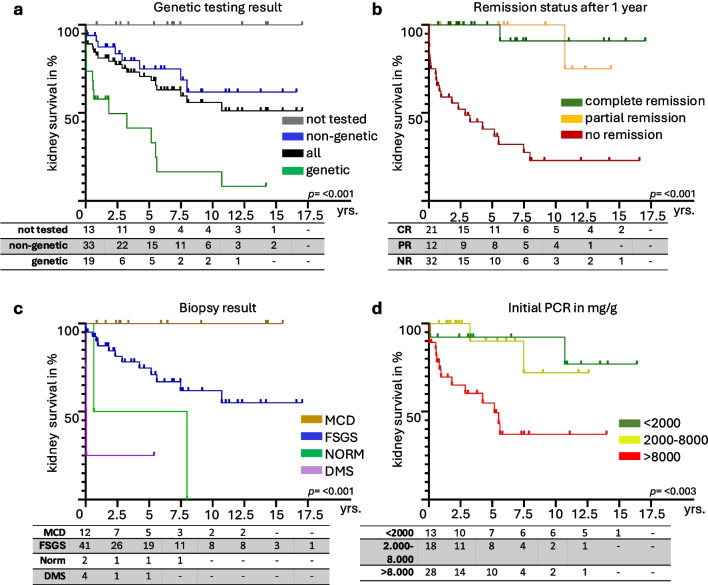
Probability of kidney survival. Stratified after (**a**) genetic testing result, (**b**) remission status after 1 year, (**c**) biopsy result, and (**d**) protein–creatinine ratio (PCR) at initial presentation in mg/g. X-axis – years of progression after initial diagnosis

Overall kidney survival rates of all patients at 5 and 10 years were 71% and 56%, respectively (Fig. 4a). At the initial presentation, 72% of patients were in CKD stage I (Supplemental Fig. 2a). This proportion decreased to 49% after 1 year and remained stable up to 5 years. At 10 years, 37% remained in CKD stage I, 5% in CKD stage II, and none in CKD stages III–IV (Supplemental Fig. 2a).

The Kaplan–Meier analysis revealed the proportion of patients with preserved kidney function at 5 and 10 years with 41% and 16% for the genetic group, 75% and 62% for the non-genetic group, and 100% for the untested group (Fig. 4a). Only 17% and 13% of patients within the genetic cohort remained in CKD stage I after 5 and 10 years respectively (Supplemental Fig. 2a). In comparison, 57% and 38% of non-genetic patients retained CKD stage I at 5 years and 10 years and 86% and 100% among the untested group (Supplemental Fig. 2a). At the last follow-up, 74% of the genetic group had progressed to kidney failure within a median of 7.4 months, compared to 27% within a median of 27.9 months in the non-genetic group (*p* < 0.001, Supplemental Fig. 2a, Table 1).

Kidney survival probability based on remission status after 1 year demonstrated that 100% or 90% in the CR group, 100% or 75% in the PR group, and 40% or 23% in the NR group at 5 or 10 years had preserved kidney function (*p* < 0.001, Fig. 4b). Only 10% of the patients who did not achieve remission after 5 years remained at CKD stage I and all patients who had not achieved remission after 10 years were in CKD stage V (Supplemental Fig. 2a). In contrast, patients who had achieved PR or CR at these time points had better kidney outcomes with no progression beyond CKD stage IIIa and most maintaining CKD stage I (Supplemental Fig. 2a).

By biopsy findings, MCD patients had 100% kidney survival at 5 and 10 years, versus 75% and 62% for FSGS and 25% at 5 years for DMS (*p* < 0.001, Fig. 4c). The level of proteinuria at initial presentation also correlated with kidney survival: PCR < 2000 mg/g had 92% survival at 5 and 10 years, PCR 2000–8000 mg/g had 90% at 5 years and 72% at 10 years, while PCR > 8000 mg/g showed significantly worse outcomes, with 55% at 5 years and 37% at 10 years (*p* = 0.003, Fig. 4d).

### Prediction factors of remission status

The likelihood of achieving remission (CR or PR) was assessed in relation to no remission (NR) using both univariate and multivariate logistic regression analyses. The univariate analysis identified several independent factors that correlated significantly with the final remission status, including consanguinity, initial proteinuria, the use of multiple immunosuppressive medications, initial eGFR, and genetic diagnosis (Table 3). The multivariate logistic regression analysis confirmed significant associations of initial PCR > 8000 mg/g (OR 0.04, *p* = 0.035) and genetic SRNS (OR 0.09, *p* = 0.030) with unfavorable remission (Table 3, Supplemental Table 3). When considering all laboratory values at specific follow-up time points in multivariate analysis, hypoalbuminemia (< 30 g/l) and eGFR at 3 months or 1 year after initial diagnosis were significantly associated with the final remission status. At 3 months, hypoalbuminemia (OR = 0.14, *p* = 0.015) and eGFR (OR = 1.03, *p* = 0.003) and at 1 year, hypoalbuminemia (OR = 0.15, *p* = 0.046) and eGFR (OR = 1.02, *p* = 0.033) correlated significantly with the final remission status (Table 3, Supplemental Table 3).

### Prediction factors of kidney survival

The risk of progressing to CKD stage V was evaluated in relation to preserved kidney function using Cox regression analysis (Table 3). In unadjusted Cox regression, early onset of disease, syndromic features, and initial eGFR were independently and significantly associated with progression to CKD stage V. Independent factors significantly correlating with kidney failure in both unadjusted and adjusted models for genetic SRNS included consanguinity (HR 4.37 95%CI 1.45–12.74, *p* = 0.007), changes to immunosuppressive treatments within the first year (HR 6.27, 95%CI 1.40–46.15, *p* = 0.032), and initial PCR > 8000 mg/g (HR 6.86, 95%CI 1.69–48.80, *p* = 0.020, Table 3). Further multiple Cox regression analysis confirmed significant association between genetic SRNS (HR 5.6, 95%CI 1.35–23.39, *p* = 0.014), initial PCR > 8000 mg/g (HR 4.98, 95%CI 1.25–25.87, *p* = 0.031), and kidney failure (Table 3). Follow-up laboratory values after 3 months and 1 year were evaluated in a multivariate Cox regression model, with and without adjustment for genetic SRNS (Table 3). At 3 months, hypoalbuminemia significantly correlated with kidney failure (HR 5.69, 95%CI 1.39–28.32, *p* = 0.020), while preserved eGFR at this point was protective (HR 0.96, 95%CI 0.93–0.98, *p* < 0.001). One year after initial presentation, hypoalbuminemia was significantly associated with kidney failure, increasing the risk nearly 50 times in the adjusted model (HR 48.3, 95%CI 4.01–1969.22, *p* = 0.010). Conversely, preserved eGFR continued to reduce the risk of kidney failure in the unadjusted model (OR 0.98, 95%CI 0.96–0.99, *p* = 0.020).

## Discussion

SRNS is a heterogeneous disease group characterized by variable kidney outcomes. In this study, we conducted a retrospective, single-center long-term study to provide additional insights into the clinical presentation, genetic profile, treatment response, and long-term kidney outcomes with a focus on potential factors influencing remission and kidney survival, in a well-defined SRNS cohort.

The median age of onset was 4 years with a slight male predominance, comparable with other studies [9, 23–25]. The median observation period was 5.85 years. At last observation, 40% of the patients were in CR, 18% in PR, and 42% in NR. These results also align with published remission outcomes [18, 24, 25]. Overall kidney survival rates were 71% at 5 years and 56% at 10 years, similar to previous published cohorts reporting 5-year survival rates of 65–84% and 10-year survival rates of 50–64% [9–11].

Within our cohort, CsA was the most applied IIS drug, followed by transition to MMF, according to current IPNA and AWMF recommendations [3, 26]. This treatment regimen was particularly successful in the non-genetic group, consistent with findings reported by others [17, 27]. SRNS patients who fail to achieve PR and lack genetic or syndromic disease should be considered for clinical trials of novel therapies according to IPNA recommendations [3]. If unavailable, CD20 cell–depleting treatment with rituximab (grade C) is advised. In our cohort, 5 patients received rituximab. For rituximab-resistant or intolerant patients, alternatives like ofatumumab and extracorporeal therapies (e.g., plasma exchange, immunoadsorption, lipid apheresis) may be considered (grade C, weak recommendation) [3]. A genetic diagnosis was established in 29% of patients, who exhibited significantly poorer kidney survival compared to those without a genetic diagnosis. In most cases, genetic testing was performed once the diagnosis of SRNS was established. The most common variants involved mutations in *NPHS2*, *NPHS1*, and *WT1*. Although monogenic SRNS is generally considered non-immune in origin, some cases demonstrated complete or partial response to immunosuppressive therapy [18, 28]. In our cohort, we observed 4 patients with genetic SRNS who achieved stabilization under CsA ± MMF treatment. Gellermann et al. and Buescher et al. reported that children with SRNS caused by *WT1* mutations responded to CsA [28, 29]. Similarly, a study of 231 SRNS patients, including 131 with monogenic forms, reported a 19% response rate to CsA treatment (3% achieving CR, 16% PR) [17], but also data from the PodoNet registry recorded a 19% response rate to immunosuppressive therapy in genetic SRNS cases [17, 18]. Different mechanisms for the immunosuppressive response in monogenic NS have been suggested: (1) the implicated genes might affect immune pathways, (2) mutations in podocytes could alter their sensitivity to immunosuppressive drugs [30, 31], or (3) corticosteroids and CsA may exert direct effects on podocytes [30].

Several studies have analyzed predictive factors for remission and kidney survival, however, often with inconclusive or imprecise results. Only a few studies have employed multivariate analysis to properly adjust for confounding effects, with Trautmann et al. being the first to include monogenic SRNS in a multivariate analysis [9, 10, 25, 32, 33]. In our cohort, we identified several predictors of unfavorable final remission status and kidney failure including early age of onset, consanguinity, syndromic features, monogenic SRNS, and use of multiple immunosuppressive regimens. Additionally, high initial proteinuria (> 8000 mg/g), low initial eGFR, and persistent hypoalbuminemia were identified as negative predictors. In fully adjusted models accounting for confounding factors, we identified monogenic disease and high initial proteinuria as well as preserved eGFR and persistent hypoalbuminemia after 3 months and 1 year as independent predictors of kidney failure and unfavorable remission status. Monogenic SRNS was the strongest predictor for NR and kidney failure, consistent with findings by Trautmann et al. [10], with consanguinity, positive family history, early age of onset, and syndromic features indicating a monogenic cause [13, 14]. While other studies have classified proteinuria in nephrotic or sub-nephrotic range, we report a more detailed stratification of initial proteinuria and demonstrate that patients with initial proteinuria above 8000 mg/g have significantly worse kidney survival outcomes. This finding aligns with observations in other glomerular diseases [34]. Moreover, eGFR and normal serum albumin levels at 3 months and 1 year were significant predictors of preserved kidney function. To our knowledge, no group has analyzed these values at early time points (especially 3 months) regarding predictive remission or kidney survival factors.

Although kidney biopsy findings significantly differed between the NR and the combined CR + PR groups (*p* < 0.001), they did not independently predict final remission status in our study. However, kidney survival rates varied significantly among different biopsy results (*p* < 0.001). The predictive value of biopsy findings for kidney failure varies between studies; while some large cohorts have demonstrated significant predictive power [10, 19, 33], others have not [9, 24, 25]. This might suggest that the underlying genetic pathophysiology, rather than the biopsy result itself, indicates disease progression.

The response to IIS has been shown to predict kidney outcomes [3, 10, 17, 23]. In our study, we also demonstrate that patients rarely change their remission status after the first year of treatment. Those who fail to respond to therapy experience significantly worse kidney outcomes, suggesting that early response to treatment may be an important predictor of long-term kidney outcomes.

Patients with genetic SRNS demonstrated a more rapid decline in kidney function, with a higher proportion progressing to CKD stage V compared to the non-genetic and untested groups. This aligns with previous studies suggesting that genetic SRNS is associated with a more aggressive disease course [10, 13, 14]. Kidney survival rates in our cohort were significantly higher in the combined non-genetic and untested group, with 80% of patients retaining preserved eGFR at the last follow-up, compared to only 26% in the genetic group. Interestingly, none of the patients without genetic testing progressed to CKD stage V. One explanation might be that some of the untested patients started treatment in countries without access to genetic screening and presented stable at all follow-ups in our clinic. Here, a genetic testing might not have been performed as no treatment adjustments were required.

The Kaplan–Meier survival analysis further supported these findings, demonstrating a more rapid decline in kidney function in the genetic group compared to the other groups. Here, our findings align with the long-term results reported by the PodoNet Registry [10, 14].

This study has several limitations, including its retrospective design and the potential for selection bias. Additionally, the absence of quantitative proteinuria measurements for some patients at initial presentation, due to prior treatment in external clinics, and the reliance on semiquantitative dipstick testing at certain follow-up points represent limitations of our study. The relatively small sample size, particularly within the genetic cohort, may limit the applicability of our findings. Additionally, 13 patients did not undergo genetic testing; however, these individuals all experienced excellent outcomes, making a genetic diagnosis unlikely. Prospective studies with larger cohorts, including genetic screening and multiple in-depth follow-up time points with long-term observation, are needed to better define prognostic factors.

In conclusion, this study highlights the heterogeneity of SRNS and the difficulties of efficient treatments, particularly in certain subgroups. In our study, genetic SRNS and high nephrotic-range proteinuria (> 8000 mg/g) at disease onset, as well as low eGFR and persistent hypoalbuminemia at early follow-up, were the most important independent predictors for unfavorable outcomes, characterized by NR and low kidney survival rates.

## Supplementary Information

Below is the link to the electronic supplementary material.

Graphical abstract (PPTX 121 KB)

Supplementary file1 (PPTX 1.06 MB)

## Data Availability

The datasets generated during and/or analyzed during the current study are available from the corresponding author on reasonable request.
