# Supplementary material for: Outcomes and prognostic factors in childhood-onset steroid-resistant nephrotic syndrome: a retrospective single-center study
Source: Pediatr Nephrol. 2025 Mar 1;40(7):2239–52. doi: 10.1007/s00467-025-06705-5 (PMC12116651; doi:10.1007/s00467-025-06705-5)
Supplement: Supplementary file 1 — Graphical abstract (PPTX 121 KB) [file 467_2025_6705_MOESM1_ESM.pptx]

## Slide 1
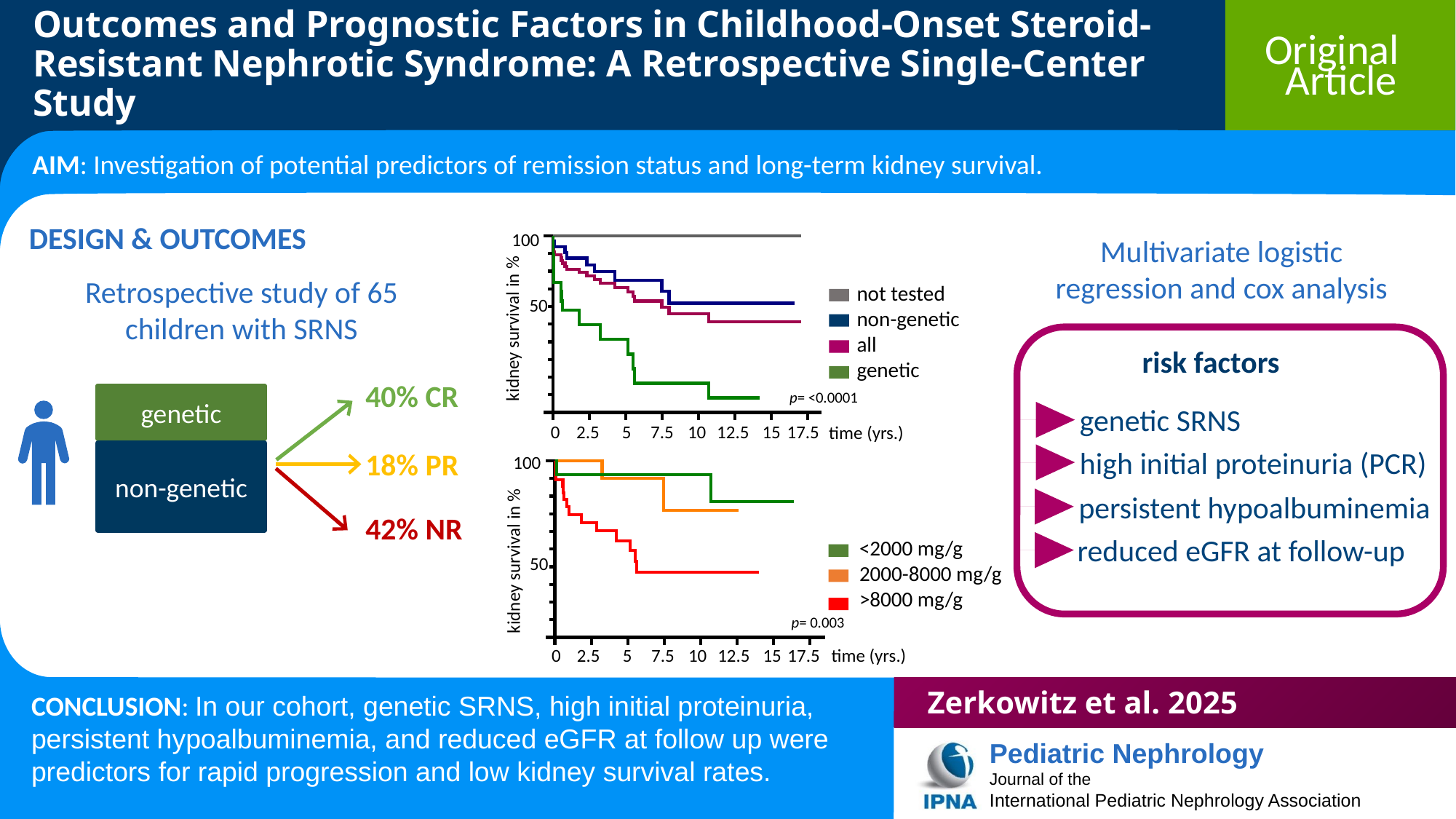

Outcomes and Prognostic Factors in Childhood-Onset Steroid-Resistant Nephrotic Syndrome: A Retrospective Single-Center Study
AIM: Investigation of potential predictors of remission status and long-term kidney survival.
DESIGN & OUTCOMES
100
Multivariate logistic regression and cox analysis
Retrospective study of 65 children with SRNS
not tested
non-genetic
all
genetic
50
kidney survival in %
risk factors
40% CR
p= <0.0001
genetic
genetic SRNS
0
2.5
5
7.5
10
12.5
15
17.5
time (yrs.)
high initial proteinuria (PCR)
18% PR
non-genetic
100
persistent hypoalbuminemia
42% NR
reduced eGFR at follow-up
<2000 mg/g
2000-8000 mg/g
>8000 mg/g
kidney survival in %
50
p= 0.003
time (yrs.)
0
2.5
5
7.5
10
12.5
15
17.5
Zerkowitz et al. 2025
CONCLUSION: In our cohort, genetic SRNS, high initial proteinuria, persistent hypoalbuminemia, and reduced eGFR at follow up were predictors for rapid progression and low kidney survival rates.
