# Supplementary material for: Outcomes and prognostic factors in childhood-onset steroid-resistant nephrotic syndrome: a retrospective single-center study
Source: Pediatr Nephrol. 2025 Mar 1;40(7):2239–52. doi: 10.1007/s00467-025-06705-5 (PMC12116651; doi:10.1007/s00467-025-06705-5)
Supplement: Supplementary file 2 — Supplementary file1 (PPTX 1.06 MB) [file 467_2025_6705_MOESM2_ESM.pptx]

## Slide 1
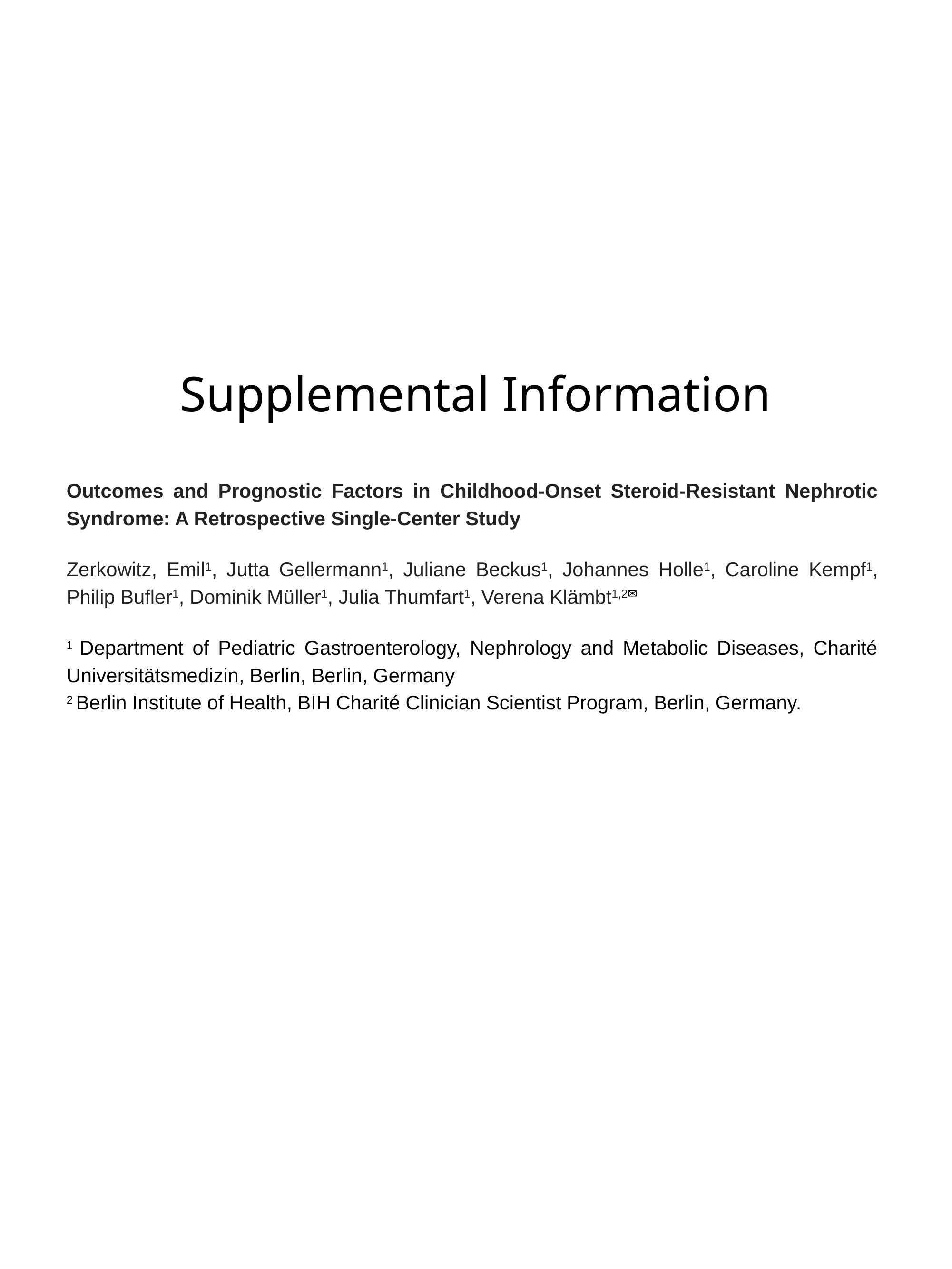

# Supplemental Information
Outcomes and Prognostic Factors in Childhood-Onset Steroid-Resistant Nephrotic Syndrome: A Retrospective Single-Center Study
Zerkowitz, Emil1, Jutta Gellermann1, Juliane Beckus1, Johannes Holle1, Caroline Kempf1, Philip Bufler1, Dominik Müller1, Julia Thumfart1, Verena Klämbt1,2✉
1 Department of Pediatric Gastroenterology, Nephrology and Metabolic Diseases, Charité Universitätsmedizin, Berlin, Berlin, Germany
2 Berlin Institute of Health, BIH Charité Clinician Scientist Program, Berlin, Germany.

## Slide 2
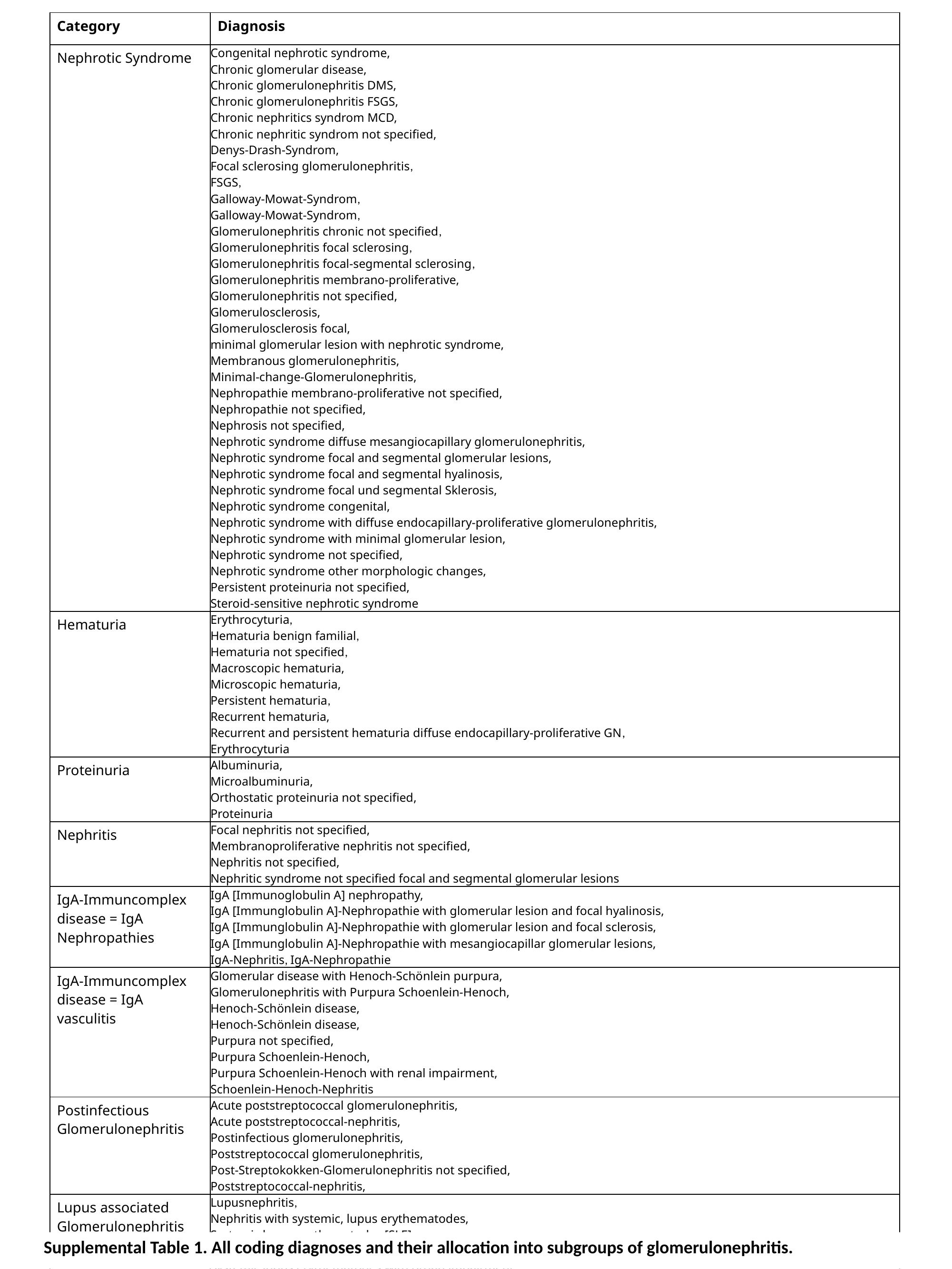

| Category | Diagnosis |
| --- | --- |
| Nephrotic Syndrome | Congenital nephrotic syndrome, Chronic glomerular disease, Chronic glomerulonephritis DMS, Chronic glomerulonephritis FSGS, Chronic nephritics syndrom MCD, Chronic nephritic syndrom not specified, Denys-Drash-Syndrom, Focal sclerosing glomerulonephritis, FSGS, Galloway-Mowat-Syndrom, Galloway-Mowat-Syndrom, Glomerulonephritis chronic not specified, Glomerulonephritis focal sclerosing, Glomerulonephritis focal-segmental sclerosing, Glomerulonephritis membrano-proliferative, Glomerulonephritis not specified, Glomerulosclerosis, Glomerulosclerosis focal, minimal glomerular lesion with nephrotic syndrome, Membranous glomerulonephritis, Minimal-change-Glomerulonephritis, Nephropathie membrano-proliferative not specified, Nephropathie not specified, Nephrosis not specified, Nephrotic syndrome diffuse mesangiocapillary glomerulonephritis, Nephrotic syndrome focal and segmental glomerular lesions, Nephrotic syndrome focal and segmental hyalinosis, Nephrotic syndrome focal und segmental Sklerosis, Nephrotic syndrome congenital, Nephrotic syndrome with diffuse endocapillary-proliferative glomerulonephritis, Nephrotic syndrome with minimal glomerular lesion, Nephrotic syndrome not specified, Nephrotic syndrome other morphologic changes, Persistent proteinuria not specified, Steroid-sensitive nephrotic syndrome |
| Hematuria | Erythrocyturia, Hematuria benign familial, Hematuria not specified, Macroscopic hematuria, Microscopic hematuria, Persistent hematuria, Recurrent hematuria, Recurrent and persistent hematuria diffuse endocapillary-proliferative GN, Erythrocyturia |
| Proteinuria | Albuminuria, Microalbuminuria, Orthostatic proteinuria not specified, Proteinuria |
| Nephritis | Focal nephritis not specified, Membranoproliferative nephritis not specified, Nephritis not specified, Nephritic syndrome not specified focal and segmental glomerular lesions |
| IgA-Immuncomplex disease = IgA Nephropathies | IgA [Immunoglobulin A] nephropathy, IgA [Immunglobulin A]-Nephropathie with glomerular lesion and focal hyalinosis, IgA [Immunglobulin A]-Nephropathie with glomerular lesion and focal sclerosis, IgA [Immunglobulin A]-Nephropathie with mesangiocapillar glomerular lesions, IgA-Nephritis, IgA-Nephropathie |
| IgA-Immuncomplex disease = IgA vasculitis | Glomerular disease with Henoch-Schönlein purpura, Glomerulonephritis with Purpura Schoenlein-Henoch, Henoch-Schönlein disease, Henoch-Schönlein disease, Purpura not specified, Purpura Schoenlein-Henoch, Purpura Schoenlein-Henoch with renal impairment, Schoenlein-Henoch-Nephritis |
| Postinfectious Glomerulonephritis | Acute poststreptococcal glomerulonephritis, Acute poststreptococcal-nephritis, Postinfectious glomerulonephritis, Poststreptococcal glomerulonephritis, Post-Streptokokken-Glomerulonephritis not specified, Poststreptococcal-nephritis, |
| Lupus associated Glomerulonephritis | Lupusnephritis, Nephritis with systemic, lupus erythematodes, Systemic lupus erythematodes [SLE], Systemic lupus erythematodes with glomerular impairment, Systemic lupus erythematodes with organ impairment, Visceral lupus erythematosus, |
| Syndromes | Hereditary Alport nephropathy, Alport syndrome, Thin basement membrane syndrome |
| ANCA | ANCA-associated vasculitis, Anca-Vasculitis, Glomerular disease with Wegener's granulomatosis |
Supplemental Table 1. All coding diagnoses and their allocation into subgroups of glomerulonephritis.

## Slide 3
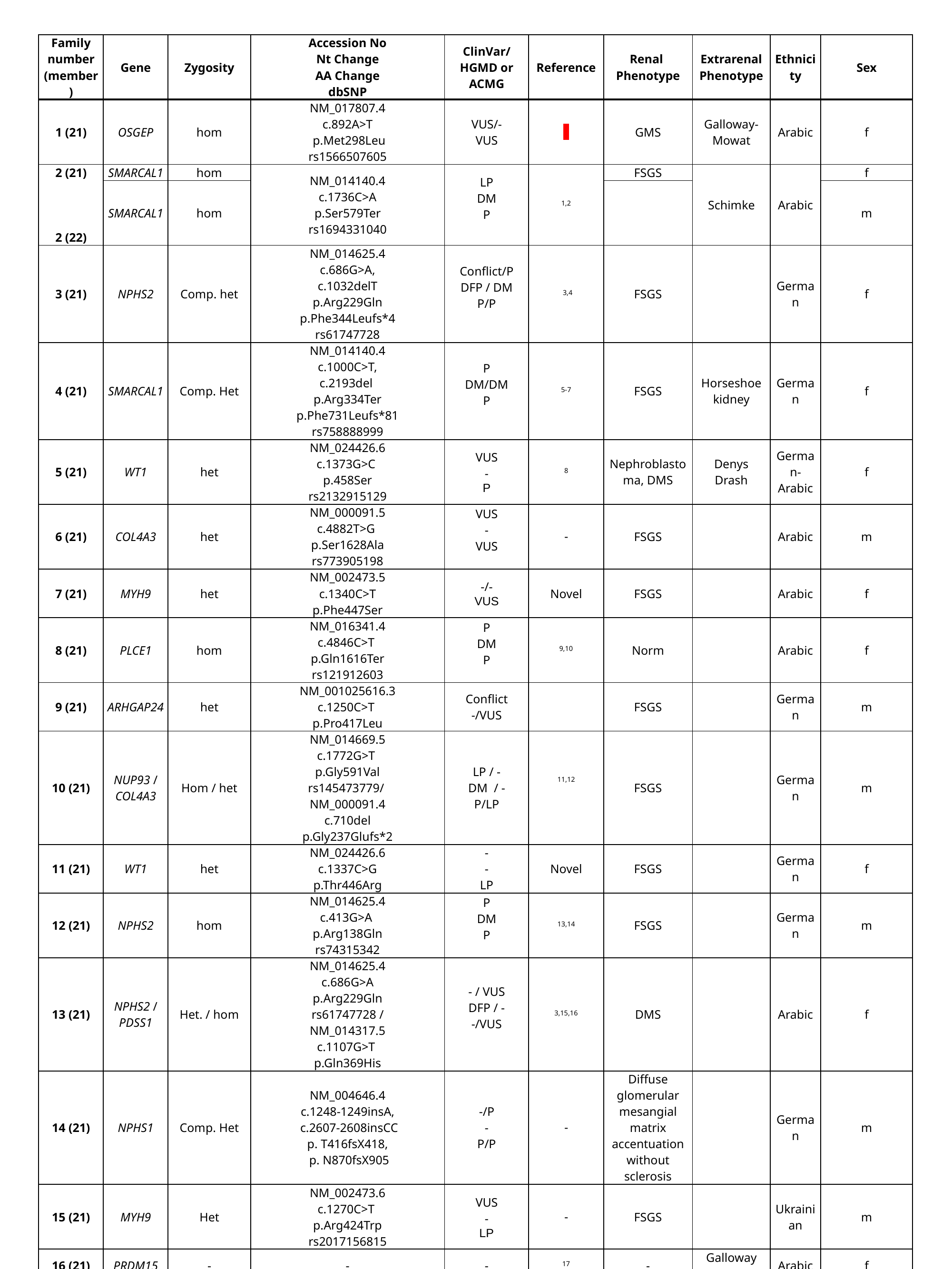

| Family number (member) | Gene | Zygosity | Accession No Nt Change AA Change dbSNP | ClinVar/HGMD or ACMG | Reference | Renal Phenotype | Extrarenal Phenotype | Ethnicity | Sex |
| --- | --- | --- | --- | --- | --- | --- | --- | --- | --- |
| 1 (21) | OSGEP | hom | NM\_017807.4 c.892A>T p.Met298Leu rs1566507605 | VUS/- VUS | | GMS | Galloway-Mowat | Arabic | f |
| 2 (21) 2 (22) | SMARCAL1 | hom | NM\_014140.4 c.1736C>A p.Ser579Ter rs1694331040 | LP DM P | 1,2 | FSGS | Schimke | Arabic | f |
| | SMARCAL1 | hom | | | | | | | m |
| 3 (21) | NPHS2 | Comp. het | NM\_014625.4 c.686G>A, c.1032delT p.Arg229Gln p.Phe344Leufs\*4 rs61747728 | Conflict/P DFP / DM P/P | 3,4 | FSGS | | German | f |
| 4 (21) | SMARCAL1 | Comp. Het | NM\_014140.4 c.1000C>T, c.2193del p.Arg334Ter p.Phe731Leufs\*81 rs758888999 | P DM/DM P | 5-7 | FSGS | Horseshoe kidney | German | f |
| 5 (21) | WT1 | het | NM\_024426.6 c.1373G>C p.458Ser rs2132915129 | VUS - P | 8 | Nephroblastoma, DMS | Denys Drash | German-Arabic | f |
| 6 (21) | COL4A3 | het | NM\_000091.5 c.4882T>G p.Ser1628Ala rs773905198 | VUS - VUS | - | FSGS | | Arabic | m |
| 7 (21) | MYH9 | het | NM\_002473.5 c.1340C>T p.Phe447Ser | -/- VUS | Novel | FSGS | | Arabic | f |
| 8 (21) | PLCE1 | hom | NM\_016341.4 c.4846C>T p.Gln1616Ter rs121912603 | P DM P | 9,10 | Norm | | Arabic | f |
| 9 (21) | ARHGAP24 | het | NM\_001025616.3 c.1250C>T p.Pro417Leu | Conflict -/VUS | | FSGS | | German | m |
| 10 (21) | NUP93 / COL4A3 | Hom / het | NM\_014669.5 c.1772G>T p.Gly591Val rs145473779/ NM\_000091.4 c.710del p.Gly237Glufs\*2 | LP / - DM / - P/LP | 11,12 | FSGS | | German | m |
| 11 (21) | WT1 | het | NM\_024426.6 c.1337C>G p.Thr446Arg | - - LP | Novel | FSGS | | German | f |
| 12 (21) | NPHS2 | hom | NM\_014625.4 c.413G>A p.Arg138Gln rs74315342 | P DM P | 13,14 | FSGS | | German | m |
| 13 (21) | NPHS2 / PDSS1 | Het. / hom | NM\_014625.4 c.686G>A p.Arg229Gln rs61747728 / NM\_014317.5 c.1107G>T p.Gln369His | - / VUS DFP / - -/VUS | 3,15,16 | DMS | | Arabic | f |
| 14 (21) | NPHS1 | Comp. Het | NM\_004646.4 c.1248-1249insA, c.2607-2608insCC p. T416fsX418, p. N870fsX905 | -/P - P/P | - | Diffuse glomerular mesangial matrix accentuation without sclerosis | | German | m |
| 15 (21) | MYH9 | Het | NM\_002473.6 c.1270C>T p.Arg424Trp rs2017156815 | VUS - LP | - | FSGS | | Ukrainian | m |
| 16 (21) | PRDM15 | - | - | - | 17 | - | Galloway Mowat | Arabic | f |

## Slide 4
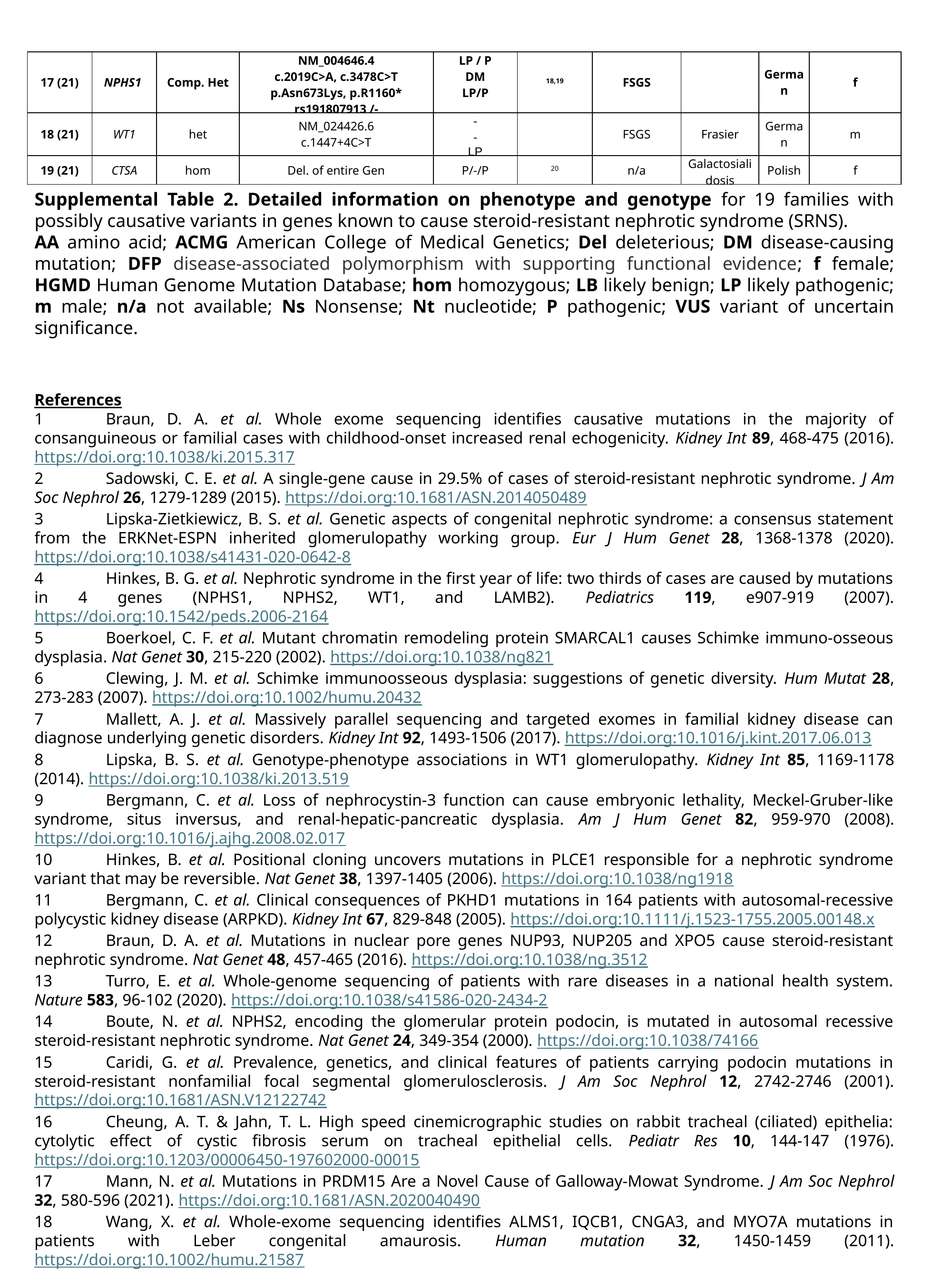

| 17 (21) | NPHS1 | Comp. Het | NM\_004646.4 c.2019C>A, c.3478C>T p.Asn673Lys, p.R1160\* rs191807913 /- | LP / P DM LP/P | 18,19 | FSGS | | German | f |
| --- | --- | --- | --- | --- | --- | --- | --- | --- | --- |
| 18 (21) | WT1 | het | NM\_024426.6 c.1447+4C>T | - - LP | | FSGS | Frasier | German | m |
| 19 (21) | CTSA | hom | Del. of entire Gen | P/-/P | 20 | n/a | Galactosialidosis | Polish | f |
Supplemental Table 2. Detailed information on phenotype and genotype for 19 families with possibly causative variants in genes known to cause steroid-resistant nephrotic syndrome (SRNS).
AA amino acid; ACMG American College of Medical Genetics; Del deleterious; DM disease-causing mutation; DFP disease-associated polymorphism with supporting functional evidence; f female; HGMD Human Genome Mutation Database; hom homozygous; LB likely benign; LP likely pathogenic; m male; n/a not available; Ns Nonsense; Nt nucleotide; P pathogenic; VUS variant of uncertain significance.
References
1	Braun, D. A. et al. Whole exome sequencing identifies causative mutations in the majority of consanguineous or familial cases with childhood-onset increased renal echogenicity. Kidney Int 89, 468-475 (2016). https://doi.org:10.1038/ki.2015.317
2	Sadowski, C. E. et al. A single-gene cause in 29.5% of cases of steroid-resistant nephrotic syndrome. J Am Soc Nephrol 26, 1279-1289 (2015). https://doi.org:10.1681/ASN.2014050489
3	Lipska-Zietkiewicz, B. S. et al. Genetic aspects of congenital nephrotic syndrome: a consensus statement from the ERKNet-ESPN inherited glomerulopathy working group. Eur J Hum Genet 28, 1368-1378 (2020). https://doi.org:10.1038/s41431-020-0642-8
4	Hinkes, B. G. et al. Nephrotic syndrome in the first year of life: two thirds of cases are caused by mutations in 4 genes (NPHS1, NPHS2, WT1, and LAMB2). Pediatrics 119, e907-919 (2007). https://doi.org:10.1542/peds.2006-2164
5	Boerkoel, C. F. et al. Mutant chromatin remodeling protein SMARCAL1 causes Schimke immuno-osseous dysplasia. Nat Genet 30, 215-220 (2002). https://doi.org:10.1038/ng821
6	Clewing, J. M. et al. Schimke immunoosseous dysplasia: suggestions of genetic diversity. Hum Mutat 28, 273-283 (2007). https://doi.org:10.1002/humu.20432
7	Mallett, A. J. et al. Massively parallel sequencing and targeted exomes in familial kidney disease can diagnose underlying genetic disorders. Kidney Int 92, 1493-1506 (2017). https://doi.org:10.1016/j.kint.2017.06.013
8	Lipska, B. S. et al. Genotype-phenotype associations in WT1 glomerulopathy. Kidney Int 85, 1169-1178 (2014). https://doi.org:10.1038/ki.2013.519
9	Bergmann, C. et al. Loss of nephrocystin-3 function can cause embryonic lethality, Meckel-Gruber-like syndrome, situs inversus, and renal-hepatic-pancreatic dysplasia. Am J Hum Genet 82, 959-970 (2008). https://doi.org:10.1016/j.ajhg.2008.02.017
10	Hinkes, B. et al. Positional cloning uncovers mutations in PLCE1 responsible for a nephrotic syndrome variant that may be reversible. Nat Genet 38, 1397-1405 (2006). https://doi.org:10.1038/ng1918
11	Bergmann, C. et al. Clinical consequences of PKHD1 mutations in 164 patients with autosomal-recessive polycystic kidney disease (ARPKD). Kidney Int 67, 829-848 (2005). https://doi.org:10.1111/j.1523-1755.2005.00148.x
12	Braun, D. A. et al. Mutations in nuclear pore genes NUP93, NUP205 and XPO5 cause steroid-resistant nephrotic syndrome. Nat Genet 48, 457-465 (2016). https://doi.org:10.1038/ng.3512
13	Turro, E. et al. Whole-genome sequencing of patients with rare diseases in a national health system. Nature 583, 96-102 (2020). https://doi.org:10.1038/s41586-020-2434-2
14	Boute, N. et al. NPHS2, encoding the glomerular protein podocin, is mutated in autosomal recessive steroid-resistant nephrotic syndrome. Nat Genet 24, 349-354 (2000). https://doi.org:10.1038/74166
15	Caridi, G. et al. Prevalence, genetics, and clinical features of patients carrying podocin mutations in steroid-resistant nonfamilial focal segmental glomerulosclerosis. J Am Soc Nephrol 12, 2742-2746 (2001). https://doi.org:10.1681/ASN.V12122742
16	Cheung, A. T. & Jahn, T. L. High speed cinemicrographic studies on rabbit tracheal (ciliated) epithelia: cytolytic effect of cystic fibrosis serum on tracheal epithelial cells. Pediatr Res 10, 144-147 (1976). https://doi.org:10.1203/00006450-197602000-00015
17	Mann, N. et al. Mutations in PRDM15 Are a Novel Cause of Galloway-Mowat Syndrome. J Am Soc Nephrol 32, 580-596 (2021). https://doi.org:10.1681/ASN.2020040490
18	Wang, X. et al. Whole-exome sequencing identifies ALMS1, IQCB1, CNGA3, and MYO7A mutations in patients with Leber congenital amaurosis. Human mutation 32, 1450-1459 (2011). https://doi.org:10.1002/humu.21587
19	Schoeb, D. S. et al. Nineteen novel NPHS1 mutations in a worldwide cohort of patients with congenital nephrotic syndrome (CNS). Nephrol Dial Transplant 25, 2970-2976 (2010). https://doi.org:10.1093/ndt/gfq088
20	Caciotti, A. et al. Galactosialidosis: review and analysis of CTSA gene mutations. Orphanet J Rare Dis 8, 114 (2013). https://doi.org:10.1186/1750-1172-8-114

## Slide 5
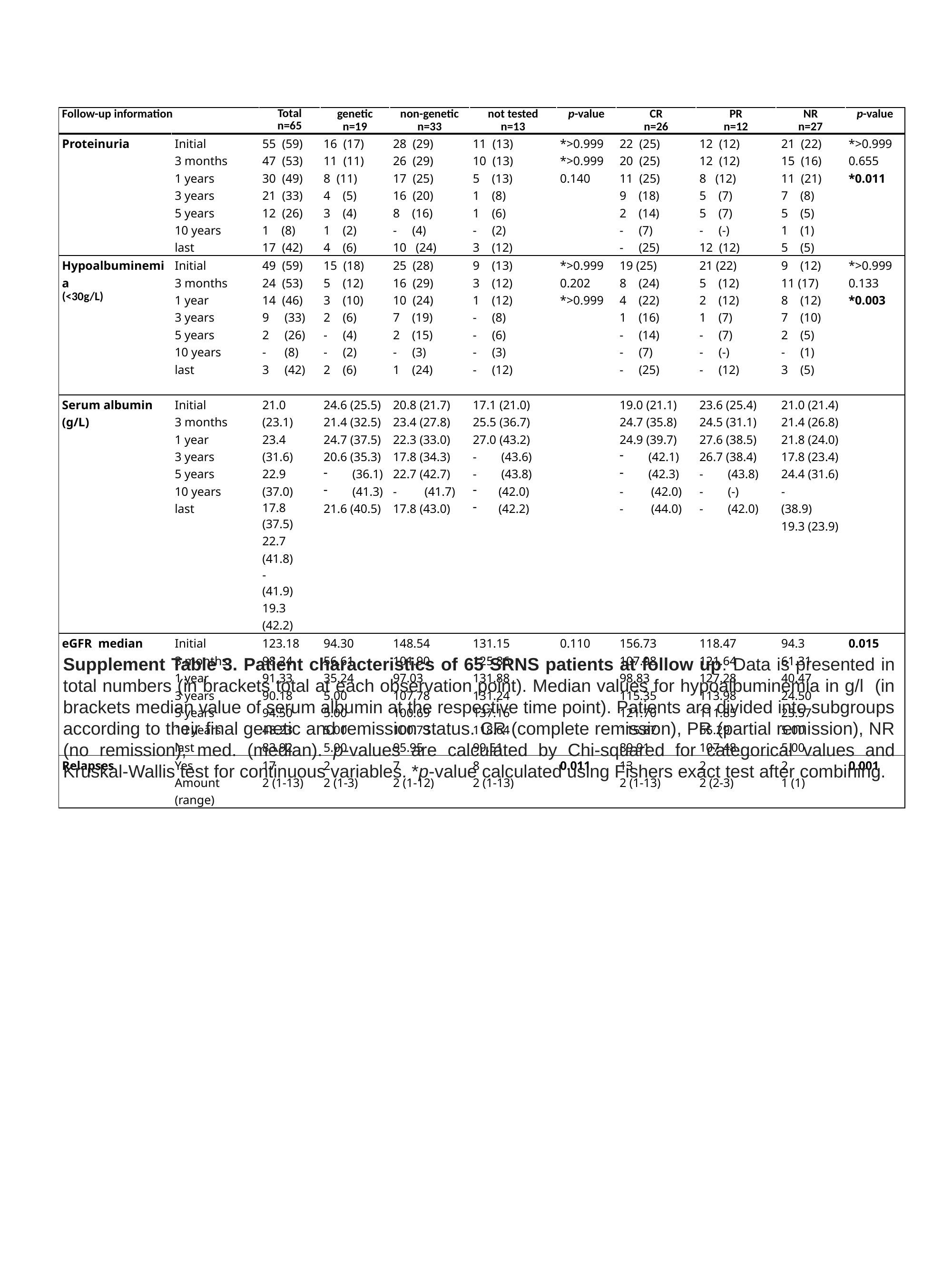

| Follow-up information | | Total n=65 | genetic n=19 | non-genetic n=33 | not tested n=13 | p-value | CR n=26 | PR n=12 | NR n=27 | p-value |
| --- | --- | --- | --- | --- | --- | --- | --- | --- | --- | --- |
| Proteinuria | Initial 3 months 1 years 3 years 5 years 10 years last | 55 (59) 47 (53) 30 (49) 21 (33) 12 (26) 1 (8) 17 (42) | 16 (17) 11 (11) 8 (11) 4 (5) 3 (4) 1 (2) 4 (6) | 28 (29) 26 (29) 17 (25) 16 (20) 8 (16) - (4) 10 (24) | 11 (13) 10 (13) 5 (13) 1 (8) 1 (6) - (2) 3 (12) | \*>0.999 \*>0.999 0.140 | 22 (25) 20 (25) 11 (25) 9 (18) 2 (14) - (7) - (25) | 12 (12) 12 (12) 8 (12) 5 (7) 5 (7) - (-) 12 (12) | 21 (22) 15 (16) 11 (21) 7 (8) 5 (5) 1 (1) 5 (5) | \*>0.999 0.655 \*0.011 |
| Hypoalbuminemia (<30g/L) | Initial 3 months 1 year 3 years 5 years 10 years last | 49 (59) 24 (53) 14  (46) 9  (33) 2   (26) -   (8) 3   (42) | 15 (18) 5 (12) 3 (10) 2 (6) - (4) - (2) 2 (6) | 25 (28) 16 (29) 10 (24) 7 (19) 2 (15) - (3) 1 (24) | 9 (13) 3  (12) 1 (12) - (8) - (6) - (3) - (12) | \*>0.999 0.202 \*>0.999 | 19 (25) 8 (24) 4 (22) 1 (16) - (14) - (7) - (25) | 21 (22) 5  (12) 2 (12) 1 (7) - (7) - (-) - (12) | 9 (12) 11 (17) 8 (12) 7 (10) 2 (5) - (1) 3 (5) | \*>0.999 0.133 \*0.003 |
| Serum albumin (g/L) | Initial 3 months 1 year 3 years 5 years 10 years last | 21.0 (23.1) 23.4 (31.6) 22.9 (37.0) 17.8 (37.5) 22.7 (41.8) - (41.9) 19.3 (42.2) | 24.6 (25.5) 21.4 (32.5) 24.7 (37.5) 20.6 (35.3) (36.1) (41.3) 21.6 (40.5) | 20.8 (21.7) 23.4 (27.8) 22.3 (33.0) 17.8 (34.3) 22.7 (42.7) - (41.7) 17.8 (43.0) | 17.1 (21.0) 25.5 (36.7) 27.0 (43.2) - (43.6) - (43.8) (42.0) (42.2) | | 19.0 (21.1) 24.7 (35.8) 24.9 (39.7) (42.1) (42.3) - (42.0) - (44.0) | 23.6 (25.4) 24.5 (31.1) 27.6 (38.5) 26.7 (38.4) - (43.8) - (-) - (42.0) | 21.0 (21.4) 21.4 (26.8) 21.8 (24.0) 17.8 (23.4) 24.4 (31.6) - (38.9) 19.3 (23.9) | |
| eGFR median | Initial 3 months 1 year 3 years 5 years 10 years last | 123.18 98.24 91.33 90.18 94.50 48.23 83.92 | 94.30 56.61 35.24 5.00 5.00 5.00 5.00 | 148.54 101.90 97.03 107.78 100.69 100.73 95.95 | 131.15 125.86 131.88 131.24 137.16 118.64 99.51 | 0.110 | 156.73 107.08 98.83 115.35 121.70 115.87 89.91 | 118.47 121.64 127.28 113.98 111.85 65.29 107.48 | 94.3 61.31 40.47 24.50 23.57 5.00 5.00 | 0.015 |
| Relapses | Yes Amount (range) | 17 2 (1-13) | 2 2 (1-3) | 7 2 (1-12) | 8 2 (1-13) | 0.011 | 13 2 (1-13) | 2 2 (2-3) | 2 1 (1) | 0.001 |
Supplement Table 3. Patient characteristics of 65 SRNS patients at follow up. Data is presented in total numbers (in brackets total at each observation point). Median values for hypoalbuminemia in g/l (in brackets median value of serum albumin at the respective time point). Patients are divided into subgroups according to their final genetic and remission status. CR (complete remission), PR (partial remission), NR (no remission), med. (median). ​p-values are calculated by Chi-squared for categorical values and Kruskal-Wallis test for continuous variables. *p-value calculated using Fishers exact test after combining.

## Slide 6
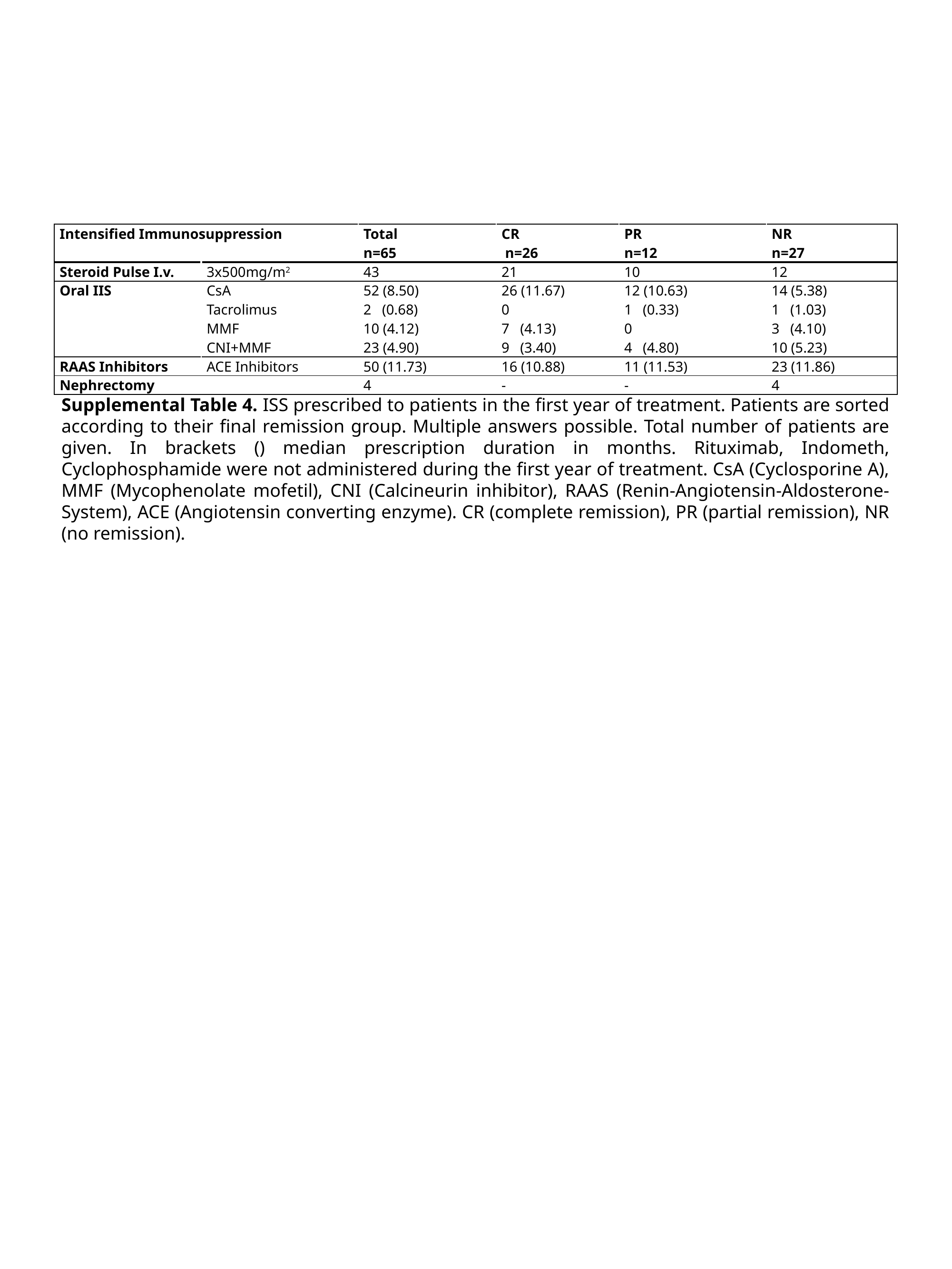

| Intensified Immunosuppression | | Total n=65 | CR n=26 | PR n=12 | NR n=27 |
| --- | --- | --- | --- | --- | --- |
| Steroid Pulse I.v. | 3x500mg/m2 | 43 | 21 | 10 | 12 |
| Oral IIS | CsA Tacrolimus MMF CNI+MMF | 52 (8.50) 2 (0.68) 10 (4.12) 23 (4.90) | 26 (11.67) 0 7 (4.13) 9 (3.40) | 12 (10.63) 1 (0.33) 0 4 (4.80) | 14 (5.38) 1 (1.03) 3 (4.10) 10 (5.23) |
| RAAS Inhibitors | ACE Inhibitors | 50 (11.73) | 16 (10.88) | 11 (11.53) | 23 (11.86) |
| Nephrectomy | | 4 | - | - | 4 |
Supplemental Table 4. ISS prescribed to patients in the first year of treatment. Patients are sorted according to their final remission group. Multiple answers possible. Total number of patients are given. In brackets () median prescription duration in months. Rituximab, Indometh, Cyclophosphamide were not administered during the first year of treatment. CsA (Cyclosporine A), MMF (Mycophenolate mofetil), CNI (Calcineurin inhibitor), RAAS (Renin-Angiotensin-Aldosterone-System), ACE (Angiotensin converting enzyme). CR (complete remission), PR (partial remission), NR (no remission).

## Slide 7
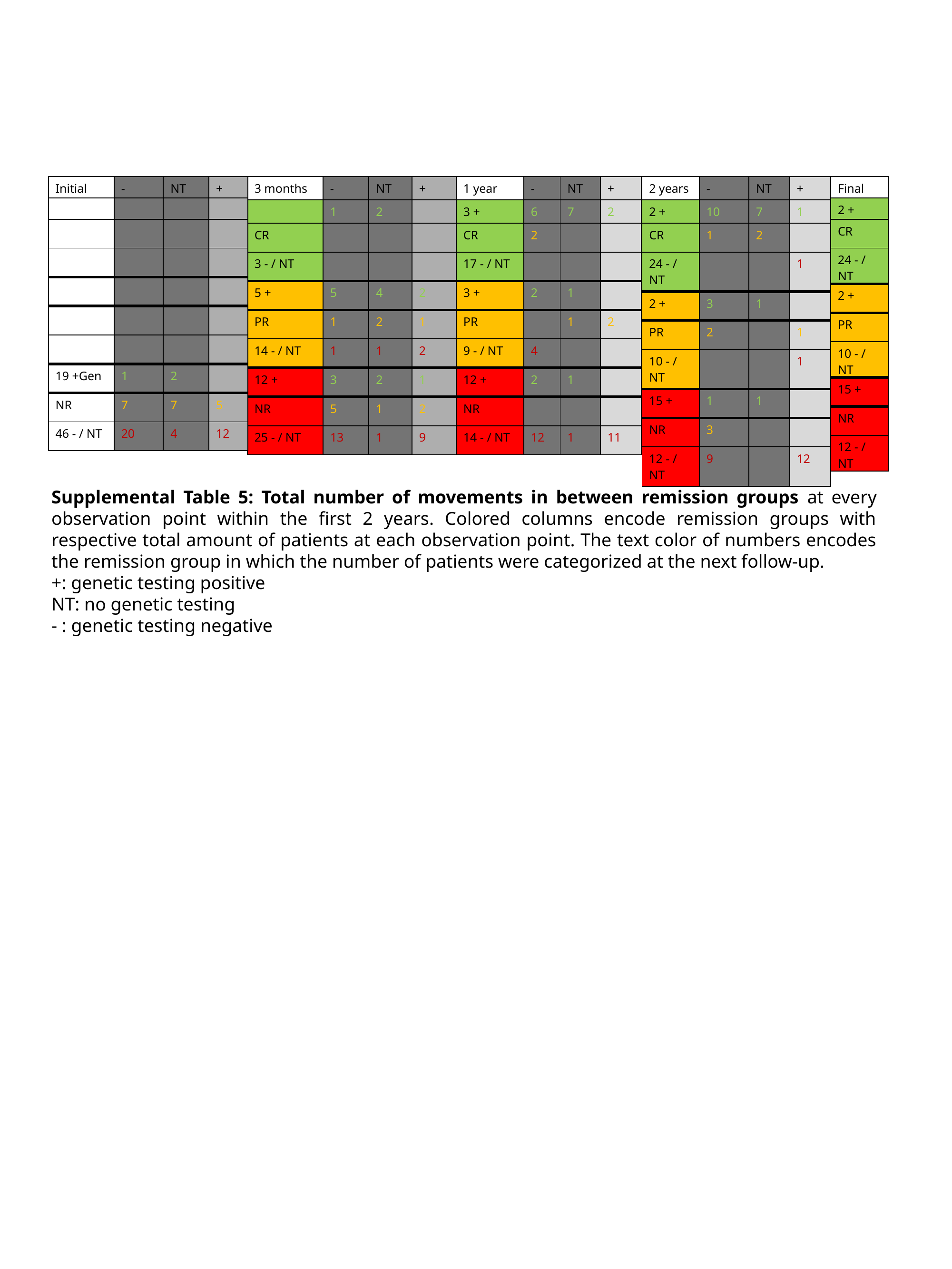

| Final |
| --- |
| 2 + |
| CR |
| 24 - / NT |
| 2 + |
| PR |
| 10 - / NT |
| 15 + |
| NR |
| 12 - / NT |
| Initial | - | NT | + |
| --- | --- | --- | --- |
| | | | |
| | | | |
| | | | |
| | | | |
| | | | |
| | | | |
| 19 +Gen | 1 | 2 | |
| NR | 7 | 7 | 5 |
| 46 - / NT | 20 | 4 | 12 |
| 3 months | - | NT | + |
| --- | --- | --- | --- |
| | 1 | 2 | |
| CR | | | |
| 3 - / NT | | | |
| 5 + | 5 | 4 | 2 |
| PR | 1 | 2 | 1 |
| 14 - / NT | 1 | 1 | 2 |
| 12 + | 3 | 2 | 1 |
| NR | 5 | 1 | 2 |
| 25 - / NT | 13 | 1 | 9 |
| 1 year | - | NT | + |
| --- | --- | --- | --- |
| 3 + | 6 | 7 | 2 |
| CR | 2 | | |
| 17 - / NT | | | |
| 3 + | 2 | 1 | |
| PR | | 1 | 2 |
| 9 - / NT | 4 | | |
| 12 + | 2 | 1 | |
| NR | | | |
| 14 - / NT | 12 | 1 | 11 |
| 2 years | - | NT | + |
| --- | --- | --- | --- |
| 2 + | 10 | 7 | 1 |
| CR | 1 | 2 | |
| 24 - / NT | | | 1 |
| 2 + | 3 | 1 | |
| PR | 2 | | 1 |
| 10 - / NT | | | 1 |
| 15 + | 1 | 1 | |
| NR | 3 | | |
| 12 - / NT | 9 | | 12 |
Supplemental Table 5: Total number of movements in between remission groups at every observation point within the first 2 years. Colored columns encode remission groups with respective total amount of patients at each observation point. The text color of numbers encodes the remission group in which the number of patients were categorized at the next follow-up.
+: genetic testing positive
NT: no genetic testing
- : genetic testing negative

## Slide 8
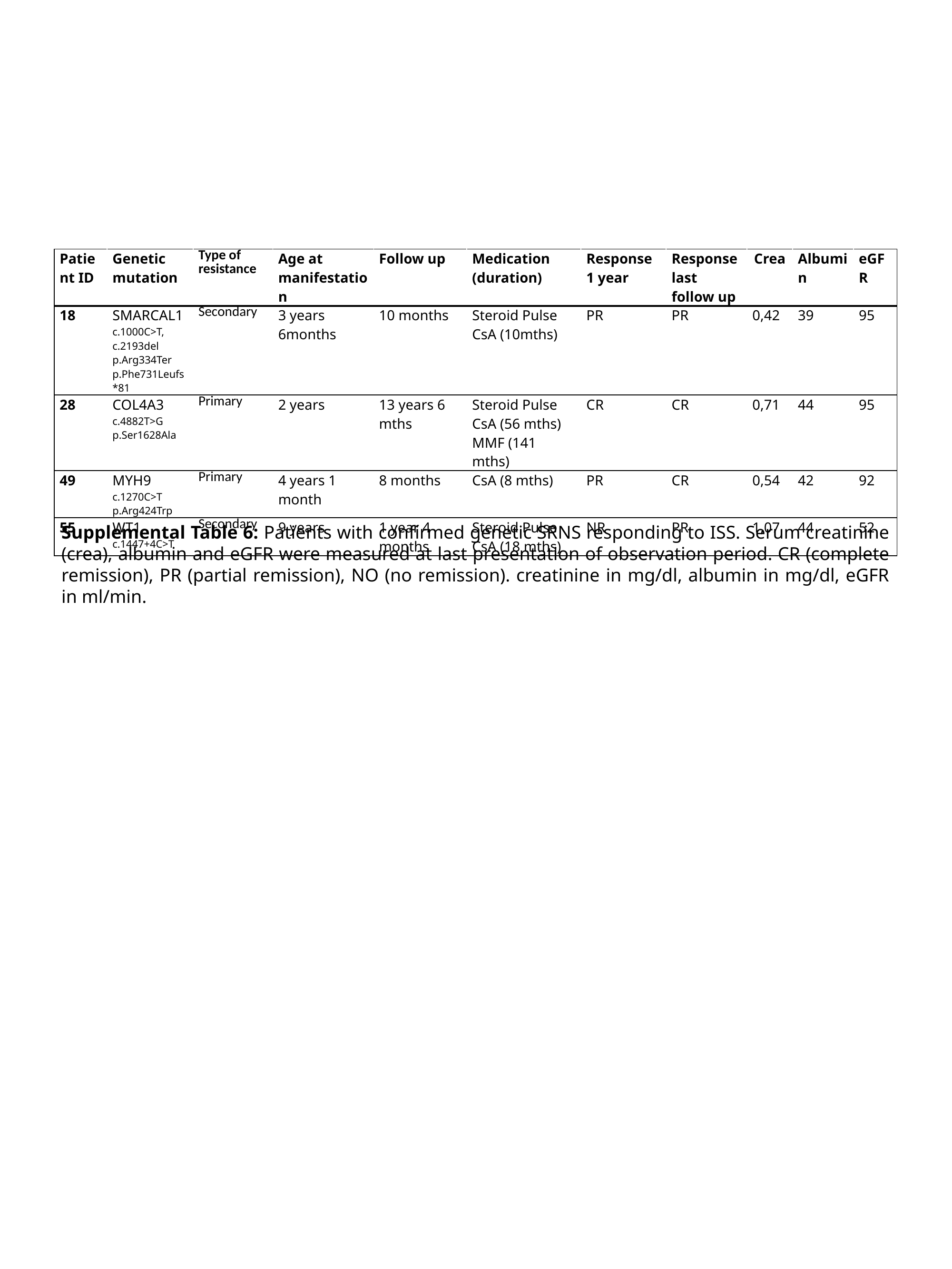

| Patient ID | Genetic mutation | Type of resistance | Age at manifestation | Follow up | Medication (duration) | Response 1 year | Response last follow up | Crea | Albumin | eGFR |
| --- | --- | --- | --- | --- | --- | --- | --- | --- | --- | --- |
| 18 | SMARCAL1 c.1000C>T, c.2193del p.Arg334Ter p.Phe731Leufs\*81 | Secondary | 3 years 6months | 10 months | Steroid Pulse CsA (10mths) | PR | PR | 0,42 | 39 | 95 |
| 28 | COL4A3 c.4882T>G p.Ser1628Ala | Primary | 2 years | 13 years 6 mths | Steroid Pulse CsA (56 mths) MMF (141 mths) | CR | CR | 0,71 | 44 | 95 |
| 49 | MYH9 c.1270C>T p.Arg424Trp | Primary | 4 years 1 month | 8 months | CsA (8 mths) | PR | CR | 0,54 | 42 | 92 |
| 55 | WT1 c.1447+4C>T | Secondary | 9 years | 1 year 4 months | Steroid Pulse CsA (18 mths) | NR | PR | 1,07 | 44 | 52 |
Supplemental Table 6: Patients with confirmed genetic SRNS responding to ISS. Serum creatinine (crea), albumin and eGFR were measured at last presentation of observation period. CR (complete remission), PR (partial remission), NO (no remission). creatinine in mg/dl, albumin in mg/dl, eGFR in ml/min.

## Slide 9
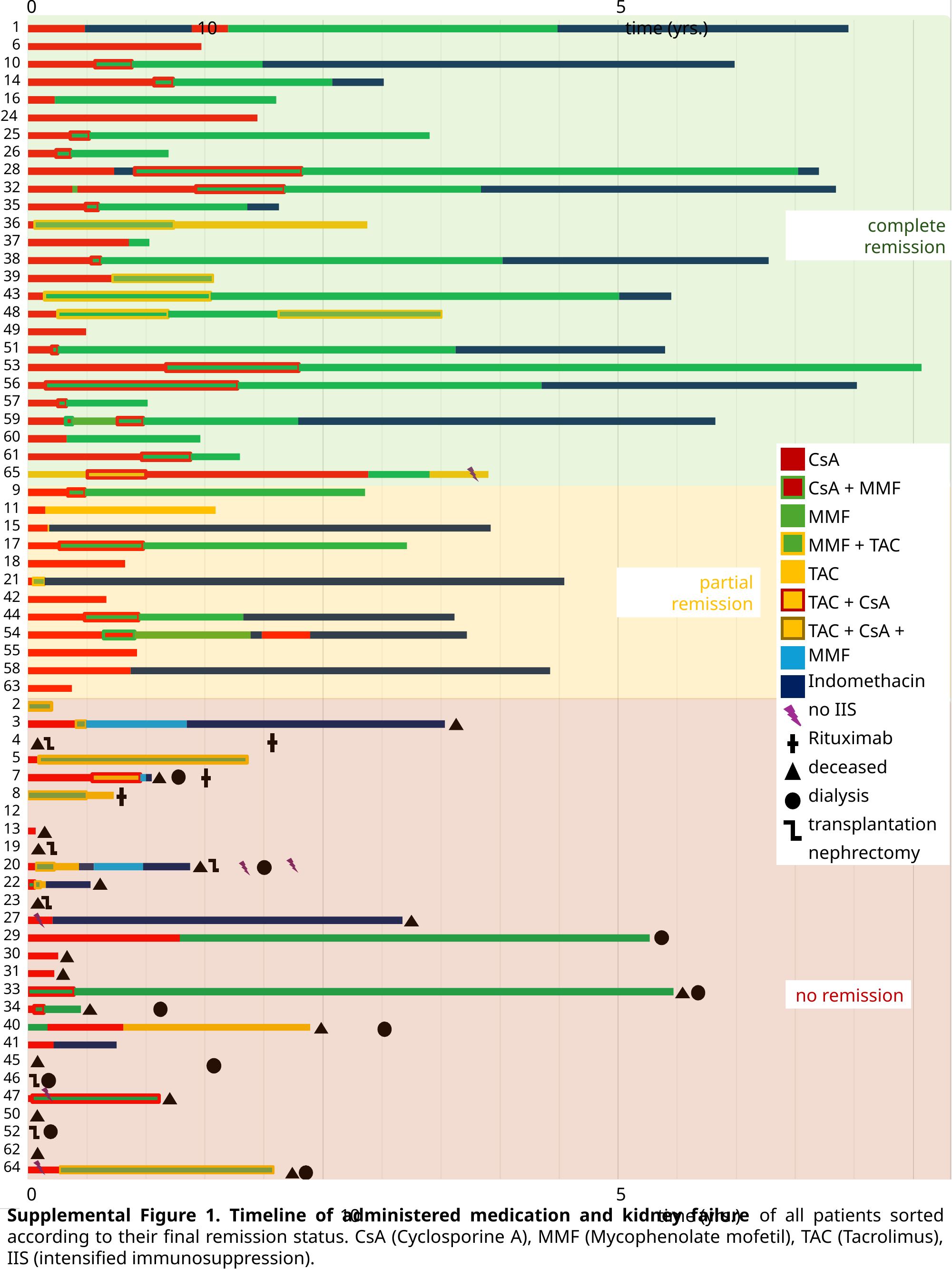

0								 5								 10				 		 time (yrs.)
1
6
10
14
16
24
25
26
28
32
35
36
37
38
39
43
48
49
51
53
56
57
59
60
61
65
9
11
15
17
18
21
42
44
54
55
58
63
2
3
4
5
7
8
12
13
19
20
22
23
27
29
30
31
33
34
40
41
45
46
47
50
52
62
64
complete remission
| | CsA |
| --- | --- |
| | CsA + MMF |
| | MMF |
| | MMF + TAC |
| | TAC |
| | TAC + CsA |
| | TAC + CsA + MMF |
| | Indomethacin |
| | no IIS |
| | Rituximab |
| | deceased |
| | dialysis |
| | transplantation |
| | nephrectomy |
partial remission
no remission
0								 5								 10				 time (yrs.)
Supplemental Figure 1. Timeline of administered medication and kidney failure of all patients sorted according to their final remission status. CsA (Cyclosporine A), MMF (Mycophenolate mofetil), TAC (Tacrolimus), IIS (intensified immunosuppression).

## Slide 10
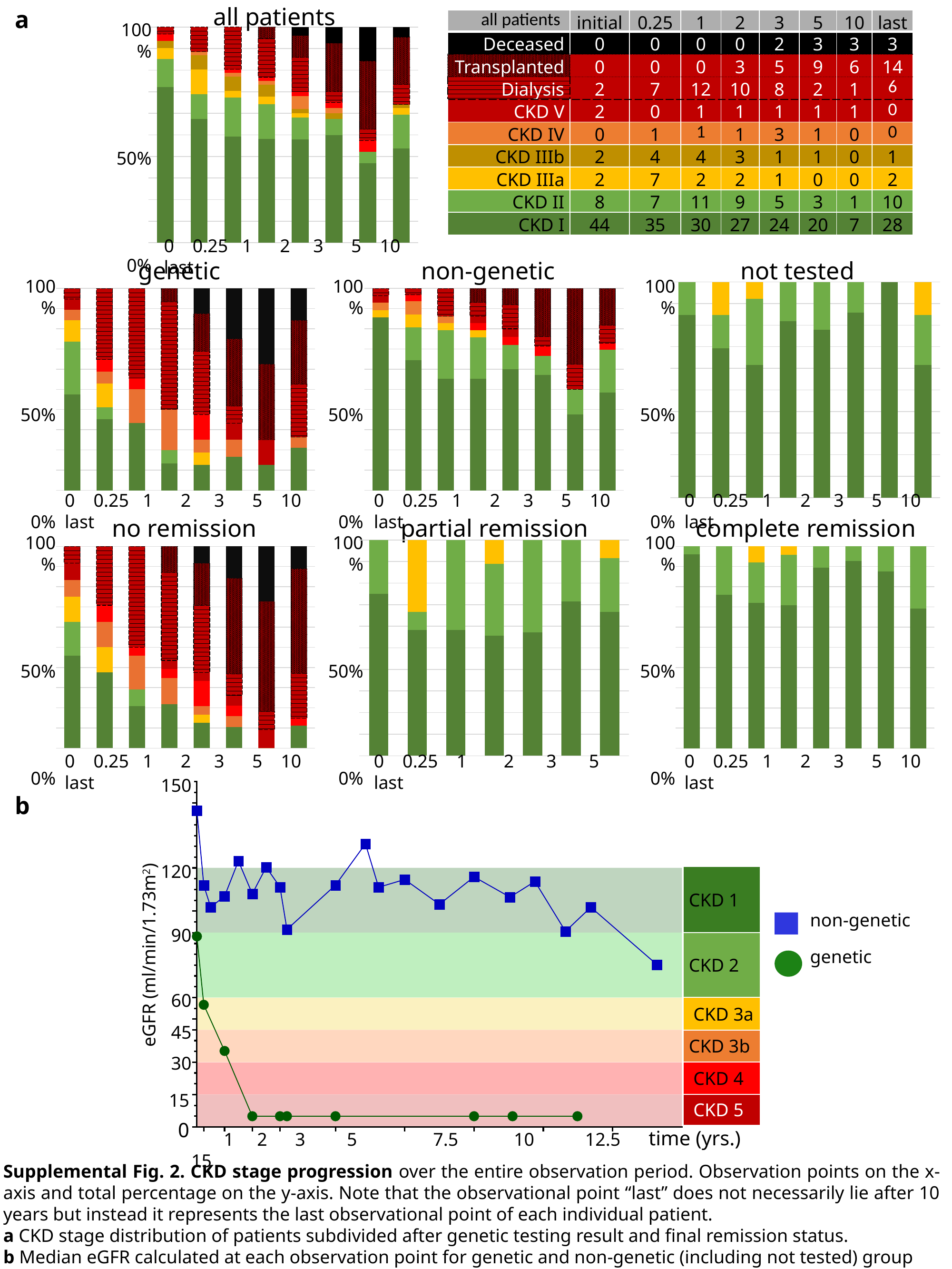

all patients
a
| all patients | initial | 0.25 | 1 | 2 | 3 | 5 | 10 | last |
| --- | --- | --- | --- | --- | --- | --- | --- | --- |
| Deceased | 0 | 0 | 0 | 0 | 2 | 3 | 3 | 3 |
| Transplanted | 0 | 0 | 0 | 3 | 5 | 9 | 6 | 14 |
| Dialysis | 2 | 7 | 12 | 10 | 8 | 2 | 1 | 6 |
| CKD V | 2 | 0 | 1 | 1 | 1 | 1 | 1 | 0 |
| CKD IV | 0 | 1 | 1 | 1 | 3 | 1 | 0 | 0 |
| CKD IIIb | 2 | 4 | 4 | 3 | 1 | 1 | 0 | 1 |
| CKD IIIa | 2 | 7 | 2 | 2 | 1 | 0 | 0 | 2 |
| CKD II | 8 | 7 | 11 | 9 | 5 | 3 | 1 | 10 |
| CKD I | 44 | 35 | 30 | 27 | 24 | 20 | 7 | 28 |
100%
50%
0%
### Chart
| Category | 1 | 2 | 3a | 3b | 4 | 5 | Dialysis | Transplanted | Deceased |
|---|---|---|---|---|---|---|---|---|---|
| 0 | 44.0 | 8.0 | 3.0 | 2.0 | 0.0 | 2.0 | 2.0 | 0.0 | 0.0 |
| 0,25 | 35.0 | 7.0 | 7.0 | 4.0 | 1.0 | 0.0 | 7.0 | 0.0 | 0.0 |
| 1 | 30.0 | 11.0 | 2.0 | 4.0 | 1.0 | 1.0 | 12.0 | 0.0 | 0.0 |
| 2 | 27.0 | 9.0 | 2.0 | 3.0 | 1.0 | 1.0 | 10.0 | 3.0 | 0.0 |
| 3 | 24.0 | 5.0 | 1.0 | 1.0 | 3.0 | 1.0 | 8.0 | 5.0 | 2.0 |
| 5 | 20.0 | 3.0 | 0.0 | 1.0 | 1.0 | 1.0 | 2.0 | 9.0 | 3.0 |
| 10 | 7.0 | 1.0 | 0.0 | 0.0 | 0.0 | 1.0 | 1.0 | 6.0 | 3.0 |
| last | 28.0 | 10.0 | 2.0 | 1.0 | 0.0 | 0.0 | 6.0 | 14.0 | 3.0 |0 0.25 1 2 3 5 10 last
genetic
non-genetic
not tested
100%
50%
0%
100%
50%
0%
100%
50%
0%
### Chart
| Category | 1 | 2 | 3a | 3b | 4 | 5 | Dialysis | Transplanted | Deceased |
|---|---|---|---|---|---|---|---|---|---|
| 0 | 9.0 | 5.0 | 2.0 | 1.0 | 0.0 | 1.0 | 1.0 | 0.0 | 0.0 |
| 0,25 | 6.0 | 1.0 | 2.0 | 1.0 | 1.0 | 0.0 | 6.0 | 0.0 | 0.0 |
| 1 | 6.0 | 0.0 | 0.0 | 3.0 | 1.0 | 0.0 | 8.0 | 0.0 | 0.0 |
| 2 | 2.0 | 1.0 | 0.0 | 3.0 | 0.0 | 0.0 | 8.0 | 1.0 | 0.0 |
| 3 | 2.0 | 0.0 | 1.0 | 1.0 | 2.0 | 0.0 | 5.0 | 3.0 | 2.0 |
| 5 | 2.0 | 0.0 | 0.0 | 1.0 | 0.0 | 1.0 | 1.0 | 4.0 | 3.0 |
| 10 | 1.0 | 0.0 | 0.0 | 0.0 | 0.0 | 1.0 | 0.0 | 3.0 | 3.0 |
| last | 4.0 | 0.0 | 0.0 | 1.0 | 0.0 | 0.0 | 5.0 | 6.0 | 3.0 |
### Chart
| Category | 1 | 2 | 3a | 3b | 4 | 5 | Dialysis | Transplanted | Deceased |
|---|---|---|---|---|---|---|---|---|---|
| 0 | 24.0 | 0.0 | 1.0 | 1.0 | 0.0 | 1.0 | 1.0 | 0.0 | 0.0 |
| 0,25 | 20.0 | 5.0 | 2.0 | 2.0 | 1.0 | 0.0 | 1.0 | 0.0 | 0.0 |
| 1 | 16.0 | 7.0 | 1.0 | 1.0 | 0.0 | 0.0 | 4.0 | 0.0 | 0.0 |
| 2 | 16.0 | 6.0 | 1.0 | 0.0 | 1.0 | 1.0 | 2.0 | 2.0 | 0.0 |
| 3 | 15.0 | 3.0 | 0.0 | 0.0 | 1.0 | 1.0 | 3.0 | 2.0 | 0.0 |
| 5 | 12.0 | 2.0 | 0.0 | 0.0 | 1.0 | 0.0 | 1.0 | 5.0 | 0.0 |
| 10 | 3.0 | 1.0 | 0.0 | 0.0 | 0.0 | 0.0 | 1.0 | 3.0 | 0.0 |
| last | 16.0 | 7.0 | 0.0 | 0.0 | 1.0 | 0.0 | 3.0 | 6.0 | 0.0 |
### Chart
| Category | 1 | 2 | 3a | 3b | 4 | 5 | Dialysis | Transplanted | Deceased |
|---|---|---|---|---|---|---|---|---|---|
| 0 | 9.0 | 5.0 | 2.0 | 1.0 | 0.0 | 1.0 | 1.0 | 0.0 | 0.0 |
| 0,25 | 6.0 | 1.0 | 2.0 | 1.0 | 1.0 | 0.0 | 6.0 | 0.0 | 0.0 |
| 1 | 6.0 | 0.0 | 0.0 | 3.0 | 1.0 | 0.0 | 8.0 | 0.0 | 0.0 |
| 2 | 2.0 | 1.0 | 0.0 | 3.0 | 0.0 | 0.0 | 8.0 | 1.0 | 0.0 |
| 3 | 2.0 | 0.0 | 1.0 | 1.0 | 2.0 | 0.0 | 5.0 | 3.0 | 2.0 |
| 5 | 2.0 | 0.0 | 0.0 | 1.0 | 0.0 | 1.0 | 1.0 | 4.0 | 3.0 |
| 10 | 1.0 | 0.0 | 0.0 | 0.0 | 0.0 | 1.0 | 0.0 | 3.0 | 3.0 |
| last | 4.0 | 0.0 | 0.0 | 1.0 | 0.0 | 0.0 | 5.0 | 6.0 | 3.0 |
### Chart
| Category | 1 | 2 | 3a | 3b | 4 | 5 | Dialysis | Transplanted | Deceased |
|---|---|---|---|---|---|---|---|---|---|
| 0 | 24.0 | 0.0 | 1.0 | 1.0 | 0.0 | 1.0 | 1.0 | 0.0 | 0.0 |
| 0,25 | 20.0 | 5.0 | 2.0 | 2.0 | 1.0 | 0.0 | 1.0 | 0.0 | 0.0 |
| 1 | 16.0 | 7.0 | 1.0 | 1.0 | 0.0 | 0.0 | 4.0 | 0.0 | 0.0 |
| 2 | 16.0 | 6.0 | 1.0 | 0.0 | 1.0 | 1.0 | 2.0 | 2.0 | 0.0 |
| 3 | 15.0 | 3.0 | 0.0 | 0.0 | 1.0 | 1.0 | 3.0 | 2.0 | 0.0 |
| 5 | 12.0 | 2.0 | 0.0 | 0.0 | 1.0 | 0.0 | 1.0 | 5.0 | 0.0 |
| 10 | 3.0 | 1.0 | 0.0 | 0.0 | 0.0 | 0.0 | 1.0 | 3.0 | 0.0 |
| last | 16.0 | 7.0 | 0.0 | 0.0 | 1.0 | 0.0 | 3.0 | 6.0 | 0.0 |
### Chart
| Category | 1 | 2 | 3a | 3b | 4 | 5 | Dialysis | Transplanted | Deceased |
|---|---|---|---|---|---|---|---|---|---|
| 0 | 11.0 | 2.0 | 0.0 | 0.0 | 0.0 | 0.0 | 0.0 | 0.0 | 0.0 |
| 0,25 | 9.0 | 2.0 | 2.0 | 0.0 | 0.0 | 0.0 | 0.0 | 0.0 | 0.0 |
| 1 | 8.0 | 4.0 | 1.0 | 0.0 | 0.0 | 0.0 | 0.0 | 0.0 | 0.0 |
| 2 | 9.0 | 2.0 | 0.0 | 0.0 | 0.0 | 0.0 | 0.0 | 0.0 | 0.0 |
| 3 | 7.0 | 2.0 | 0.0 | 0.0 | 0.0 | 0.0 | 0.0 | 0.0 | 0.0 |
| 5 | 6.0 | 1.0 | 0.0 | 0.0 | 0.0 | 0.0 | 0.0 | 0.0 | 0.0 |
| 10 | 3.0 | 0.0 | 0.0 | 0.0 | 0.0 | 0.0 | 0.0 | 0.0 | 0.0 |
| last | 8.0 | 3.0 | 2.0 | 0.0 | 0.0 | 0.0 | 0.0 | 0.0 | 0.0 |0 0.25 1 2 3 5 10 last
0 0.25 1 2 3 5 10 last
0 0.25 1 2 3 5 10 last
no remission
partial remission
complete remission
100%
50%
0%
100%
50%
0%
100%
50%
0%
### Chart
| Category | 1 | 2 | 3a | 3b | 4 | 5 | Dialysis | Transplanted | Deceased |
|---|---|---|---|---|---|---|---|---|---|
| 0 | 11.0 | 4.0 | 3.0 | 2.0 | 0.0 | 2.0 | 2.0 | 0.0 | 0.0 |
| 0,25 | 9.0 | 0.0 | 3.0 | 3.0 | 2.0 | 0.0 | 7.0 | 0.0 | 0.0 |
| 1 | 5.0 | 2.0 | 0.0 | 4.0 | 1.0 | 0.0 | 12.0 | 0.0 | 0.0 |
| 2 | 5.0 | 0.0 | 0.0 | 3.0 | 1.0 | 1.0 | 10.0 | 3.0 | 0.0 |
| 3 | 3.0 | 0.0 | 1.0 | 1.0 | 3.0 | 1.0 | 8.0 | 5.0 | 2.0 |
| 5 | 2.0 | 0.0 | 0.0 | 1.0 | 1.0 | 1.0 | 2.0 | 9.0 | 3.0 |
| 10 | 0.0 | 0.0 | 0.0 | 0.0 | 0.0 | 1.0 | 1.0 | 6.0 | 3.0 |
| last | 3.0 | 0.0 | 0.0 | 0.0 | 1.0 | 0.0 | 6.0 | 14.0 | 3.0 |
### Chart
| Category | 1 | 2 | 3a | 3b | 4 | 5 | Dialysis | Transplanted | Deceased |
|---|---|---|---|---|---|---|---|---|---|
| 0 | 9.0 | 3.0 | 0.0 | 0.0 | 0.0 | 0.0 | 0.0 | 0.0 | 0.0 |
| 0,25 | 7.0 | 1.0 | 4.0 | 0.0 | 0.0 | 0.0 | 0.0 | 0.0 | 0.0 |
| 1 | 7.0 | 5.0 | 0.0 | 0.0 | 0.0 | 0.0 | 0.0 | 0.0 | 0.0 |
| 2 | 5.0 | 3.0 | 1.0 | 0.0 | 0.0 | 0.0 | 0.0 | 0.0 | 0.0 |
| 3 | 4.0 | 3.0 | 0.0 | 0.0 | 0.0 | 0.0 | 0.0 | 0.0 | 0.0 |
| 5 | 5.0 | 2.0 | 0.0 | 0.0 | 0.0 | 0.0 | 0.0 | 0.0 | 0.0 |
| last | 8.0 | 3.0 | 1.0 | 0.0 | 0.0 | 0.0 | 0.0 | 0.0 | 0.0 |
### Chart
| Category | 1 | 2 | 3a | 3b | 4 | 5 | Dialysis | Transplanted | Deceased |
|---|---|---|---|---|---|---|---|---|---|
| 0 | 24.0 | 1.0 | 0.0 | 0.0 | 0.0 | 0.0 | 0.0 | 0.0 | 0.0 |
| 0,25 | 19.0 | 6.0 | 0.0 | 0.0 | 0.0 | 0.0 | 0.0 | 0.0 | 0.0 |
| 1 | 18.0 | 5.0 | 2.0 | 0.0 | 0.0 | 0.0 | 0.0 | 0.0 | 0.0 |
| 2 | 17.0 | 6.0 | 1.0 | 0.0 | 0.0 | 0.0 | 0.0 | 0.0 | 0.0 |
| 3 | 17.0 | 2.0 | 0.0 | 0.0 | 0.0 | 0.0 | 0.0 | 0.0 | 0.0 |
| 5 | 13.0 | 1.0 | 0.0 | 0.0 | 0.0 | 0.0 | 0.0 | 0.0 | 0.0 |
| 10 | 7.0 | 1.0 | 0.0 | 0.0 | 0.0 | 0.0 | 0.0 | 0.0 | 0.0 |
| last | 18.0 | 8.0 | 0.0 | 0.0 | 0.0 | 0.0 | 0.0 | 0.0 | 0.0 |0 0.25 1 2 3 5 10 last
0 0.25 1 2 3 5 last
0 0.25 1 2 3 5 10 last
150
b
120
| CKD 1 |
| --- |
| CKD 2 |
| CKD 3a |
| CKD 3b |
| CKD 4 |
| CKD 5 |
non-genetic
90
eGFR (ml/min/1.73m2)
genetic
60
45
30
15
0
time (yrs.)
  1   2 3 5	 7.5 10 12.5 15
Supplemental Fig. 2. CKD stage progression over the entire observation period. Observation points on the x-axis and total percentage on the y-axis. Note that the observational point “last” does not necessarily lie after 10 years but instead it represents the last observational point of each individual patient.
a CKD stage distribution of patients subdivided after genetic testing result and final remission status.
b Median eGFR calculated at each observation point for genetic and non-genetic (including not tested) group
